# The Real Bounty: Marine Biodiversity in the Pitcairn Islands

**DOI:** 10.1371/journal.pone.0100142

**Published:** 2014-06-25

**Authors:** Alan M. Friedlander, Jennifer E. Caselle, Enric Ballesteros, Eric K. Brown, Alan Turchik, Enric Sala

**Affiliations:** 1 Pristine Seas, National Geographic Society, Washington DC, United States of America; 2 Fisheries Ecology Research Laboratory, Department of Biology, University of Hawaii, Honolulu, Hawaii, United States of America; 3 Marine Science Institute, University of California Santa Barbara, Santa Barbara, California, United States of America; 4 Centre d'Estudis Avançats de Blanes, Blanes, Spain; 5 Kalaupapa National Historical Park, US National Park Service, Kalaupapa, Hawaii, United States of America; Technical University of Denmark, Denmark

## Abstract

In 2012 we conducted an integrated ecological assessment of the marine environment of the Pitcairn Islands, which are four of the most remote islands in the world. The islands and atolls (Ducie, Henderson, Oeno, and Pitcairn) are situated in the central South Pacific, halfway between New Zealand and South America. We surveyed algae, corals, mobile invertebrates, and fishes at 97 sites between 5 and 30 m depth, and found 51 new records for algae, 23 for corals, and 15 for fishes. The structure of the ecological communities was correlated with age, isolation, and geomorphology of the four islands. Coral and algal assemblages were significantly different among islands with Ducie having the highest coral cover (56%) and Pitcairn dominated by erect macroalgae (42%). Fish biomass was dominated by top predators at Ducie (62% of total fish biomass) and at Henderson (35%). Herbivorous fishes dominated at Pitcairn, while Oeno showed a balanced fish trophic structure. We found high levels of regional endemism in the fish assemblages across the islands (45%), with the highest level observed at Ducie (56% by number). We conducted the first surveys of the deep habitats around the Pitcairn Islands using drop-cameras at 21 sites from depths of 78 to 1,585 m. We observed 57 fish species from the drop-cams, including rare species such as the false catshark (*Pseudotriakis microdon*) and several new undescribed species. In addition, we made observations of typically shallow reef sharks and other reef fishes at depths down to 300 m. Our findings highlight the uniqueness and high biodiversity value of the Pitcairn Islands as one of the least impacted in the Pacific, and suggest the need for immediate protection.

## Introduction

**Figure 1:**
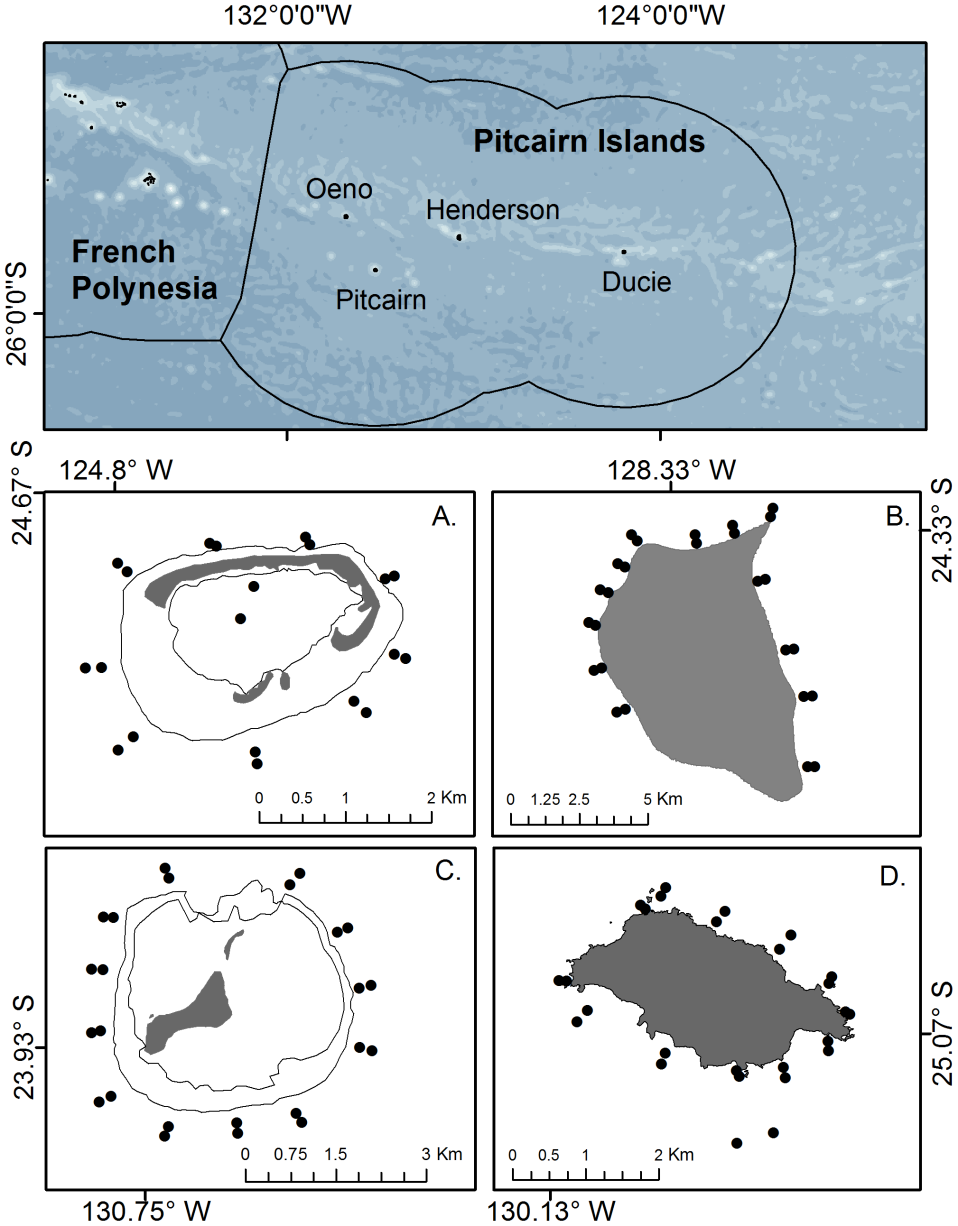
The Pitcairn Islands Exclusive Economic Zone (EEZ) covers ca. 836,108 km^2^ and encompasses two coral atolls (Ducie [A.] and Oeno [C.]), a raised atoll (Henderson [B.]), and one high island (Pitcairn [D.]). Black dots represent sampling locations around each island.

Pitcairn Island is perhaps best known as the home of the descendants of the infamous HMS Bounty mutineers [1]–[2], and is the last remaining British Overseas Territory in the Pacific [3]–[4]. The Pitcairn Islands consist of four remote islands and atolls (Ducie, Henderson, Oeno, and Pitcairn), situated in the central South Pacific, with the closest islands being the Gambier Group in French Polynesia, 390 km to the west. To the east, only Easter Island (1,900 km away) and Salas y Gómez (2,300 km) can be found between Ducie and South America [5]. Together, all four islands encompass only 43 km^2^ of emergent land, but the surrounding waters out to the 200 nautical mile Exclusive Economic Zone (EEZ) cover ca. 836,108 km^2^ (Fig. 1, [6]).

Of the four islands, only Pitcairn is inhabited, with a current population of 53 people [7]–[8]. Although the history of the human occupation of Pitcairn – and to a lesser extent Henderson – has received enormous attention [1], [9]–[11], relatively little is known about its natural history, especially with regard to the marine environment. Until our expedition in 2012, only lists of species and qualitative estimates of abundance were available for major groups of marine organisms [12]–[17], and no quantitative assessments of these populations had been conducted (see review [18] for a list of the expeditions conducted in Pitcairn's EEZ).

Because of its relatively high latitude and distance away from the Coral Triangle – the center of marine biodiversity [19]–[20] – the Pitcairn Islands have relatively low species richness for most marine taxa [12], [17]–[18]. This isolation and their subtropical location, however, make them interesting from a biogeographic perspective as they lie at the eastern limits of the Indo-Pacific Province [21]–[22]. In addition, remote locations with minimal human impacts are some of the last remaining places on earth where we can observe how coral reefs may have functioned in the distant past, before extensive human disturbance [23]–[24].

Here we present the first quantitative data on the community structure of shallow marine ecosystems of the Pitcairn Islands. Our surveys were designed to measure the abundance and biomass of major organisms (including algae, invertebrates, and fishes) inhabiting the coral reef ecosystems to 30 m depth, and to construct the first list of the deep-sea species to 1,600 m depth. The overall objective of this integrated assessment was to quantitatively describe the structure and function of the marine ecosystem of this remote group of islands, and establish a baseline for future comparisons.

## Methods

### Ethics Statement

The Government of the United Kingdom and the Pitcairn Island Council granted all necessary permission to conduct this research. No vertebrate sampling was conducted and therefore no approval was required by the Institutional Animal Care and Use Committee.

### Location

The Pitcairn Islands are the only emergent parts of an ancient chain of volcanoes that rose from the seafloor between 0.9 and 16 Myr ago [25], and are geologically connected to the Tuamotu and Gambier islands of French Polynesia [26]. The four islands differ in their size, geological age, and isolation [27]. Pitcairn is a high volcanic island of 450 ha with lava cliffs and rugged hills rising to a peak at 335 m. Henderson (200 km ENE of Pitcairn) is the largest island in the group with an area of 4,310 ha. Henderson was formerly an atoll, but the formation of Pitcairn 0.8–0.9 Myr ago caused an uplift of the crust, which elevated Henderson 33 m above sea level [28]. Henderson was declared a UNESCO World Heritage site owing to its unique terrestrial natural history and ecological intactness [29]. Ducie (472 km E of Pitcairn), the most southerly coral atoll in the world [30], consists of a central lagoon surrounded by four islets covering 70 ha. Oeno (120 km NW of Pitcairn) is a low coral atoll of 65 ha comprising a central low-lying island surrounded by a shallow lagoon and fringing reef (diameter ca. 4 km).

### Sample Design

Sampling sites were haphazardly selected around all four islands to incorporate representative wave exposures, habitats, and oceanographic conditions (Fig. 1). At each site, SCUBA surveys were conducted at both 10 and 20 m depth. In addition, two sites at 30 m were surveyed at Pitcairn to characterize the deeper reef community, as well as surveys conducted on the patch reefs (∼5 m) in the shallow lagoon of Ducie.

### Benthic communities

Characterization of the benthos was conducted along a 50 m-long transect parallel to the shoreline at each sampling depth strata. For algae, corals, and other sessile invertebrates we used a line-point intercept methodology along transects, recording the species or taxa found every 20 cm on the measuring tape. Point contact data were expressed as percent cover. For sea urchins, we counted and sized individuals in fifteen, 50×50 cm quadrats haphazardly placed along each 50 m transect line. Quadrat placement was stratified with three quadrats per 10 m segment of transect line.

### Reef fishes

At each depth stratum within a site, one diver counted and estimated lengths for all fishes encountered within fixed-length (25-m) belt transects whose widths differed depending on direction of swim. Transect bearings were set along isobaths within homogeneous habitats with each transect separated by at least 5 m. All fish ≥20 cm total length (TL) were tallied within a 4 m wide strip surveyed on an initial “swim-out” as the transect line was laid (transect area  = 100 m^2^). These included large-bodied, vagile fishes. All fishes <20 cm TL were tallied within a 2 m wide strip surveyed on the return swim back along the laid transect line (transect area  = 50 m^2^). This included small-bodied, less vagile and more site-attached fish. In addition, all species observed outside of the transect area at each station were recorded to estimate total species richness at a site.

Fishes were identified to species [31]. Fish total length (TL) was estimated to the nearest cm and individual-specific lengths were converted to body weights. Numerical density (abundance) was expressed as number of individuals per m^2^ and biomass density was expressed as tons per ha. The biomass of individual fishes was estimated using the allometric length-weight conversion: W = aTL^b^, where parameters a and b are species-specific constants, TL is total length in cm, and W is weight in grams. Length-weight fitting parameters were obtained from FishBase [32] and other published sources [33], [34]. The cross-product of individual weights and numerical densities was used to estimate biomass density by species. Fishes were categorized into four trophic groups (top predators, herbivores, other carnivores, and planktivores) after [35]–[36].

### Deep drop-camera surveys

National Geographic's Remote Imaging Team developed deep ocean drop-cams, which are high definition cameras (Sony Handycam HDR-XR520V 12 megapixel) encased in a borosilicate glass sphere that are rated to 10,000 m depth. Viewing area per frame was between 2–6 m^2^, depending on the steepness of the slope where the drop-cam landed. Cameras were baited with frozen fish and deployed for ca. four hours. The cameras remained sealed during the entire expedition with communications through a Subconn connector. Lighting at depth was achieved through a high intensity LED array directed using external reflectors. Depth gauging was conducted using an external pressure sensor. The drop-cams were ballasted with a 22 kg external weight that resulted in a descent rate of 1.5 m s^−1^. The primary release mechanism was a burn wire that was activated using onboard battery voltage. The drop-cams are positively buoyant resulting in an ascent rate of 0.5 m s^−1^. Drop-cams have an onboard VHF transmitter that allows for recovery using locating antennae with backup location achieved via communication with the ARGOS satellite system.

### Statistical analyses

Percent substrate cover for each major functional group (corals [included the cnidarian orders Anthomedusae, Alcyonacea, Scleractinia, and Zoantharia], crustose coralline algae [CCA], erect macroalgae, turf algae, dead coral + rock [DCR], and sand) was derived for each site. Sites were stratified by depth (10, 20 m) with the 30 m sites at Pitcairn and the 5 m patch reef sites at Ducie excluded from comparisons among islands.

Correlation between geological age of the islands and coral species richness was tested with a Pearson Product Moment Correlation (α = 0.05). Differences in percent substrate cover of the four dominant functional groups (CCA, coral, erect macroalgae, and turf algae) were tested among islands and between depths using multivariate analysis of variance (MANOVA). These four primary habitat functional groups comprised over 85% of the total cover and were arcsine square root transformed prior to statistical analysis to conform to the assumptions of the MANOVA. The multivariate test statistic Pillai's trace was used because it is robust to heterogeneity of variance and is less likely to involve type I errors than comparable tests [37]. We performed univariate ANOVAs when MANOVAs were significant. Unplanned comparisons between pairs of islands were examined using the Tukey-Kramer honestly significant difference (HSD) test for ANOVAs (α = 0.05).

Non-metric multi-dimensional scaling (nMDS) analysis, coupled with an analysis of similarities (ANOSIM) test, was conducted using PRIMER v6 [38] to examine differences in benthic communities and fish assemblages between islands and depth strata. Separate Bray–Curtis similarity matrices were created for percent cover of algae by species, percent cover of coral by species, sea urchin species density (no. m^−2^), and fish biomass in t ha^−1^ by species for each site and depth. Prior to conducting the nMDS, algal and coral data percentage data were arcsin square root transformed, while sea urchin density and fish biomass data were square root transformed. ANOSIM analysis generates an R statistic that scales from 0 or negative value (identical assemblages) to 1 (completely dissimilar assemblages). The resulting P value indicates the probability that the two assemblages come from a similar distribution [39]. Pairwise ANOSIM R statistics represent comparisons that are well separated (R>0.75), overlapping but clearly different (R>0.5), or barely separable at all (R<0.25). A two-way crossed ANOSIM with replication was used to compare between island and depth strata. A Bray–Curtis similarity matrix was created from the arcsin square root transformed percentage benthic cover and square root transformed mean fish biomass matrix before conducting the nMDS. The nMDS plot overlaid the primary species vectors driving the ordination using a Pearson correlation at p>0.5.

Fish species richness was estimated as the total number of species observed per station. Species diversity was calculated from the Shannon-Weaver Diversity Index [40]: 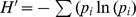, where p_i_ is the proportion of all individuals counted that were of species i. Fish assemblage characteristics among islands were compared using two-way analysis of variance (ANOVA) by island and depth strata. Numerical abundance and biomass were ln(x+1)-transformed prior to statistical analysis to conform to the assumptions of the parametric tests [41]. Normality was tested using a Shapiro-Wilk W test (P>0.05) while a Bartlett's test (P>0.05) was used to examine homogeneity of variance. Unplanned comparisons between pairs were examined using the Tukey-Kramer HSD.

To describe the pattern of variation in community structure (patterns of distribution of abundance of functional groups within the community) among the four islands, we used indirect gradient analysis. Non-linear models were most appropriate for our data because a preliminary detrended correspondence analysis showed long gradient lengths (>2 SD) [42]. To explore the spatial distribution of community structure across the archipelago we performed a correspondence analysis (CA) [42] on log-transformed data using the ordination program CANOCO for Windows version 4.0 [43]. We pooled data from all taxa into the following groups to facilitate the large-scale analysis: biomass of the four fish trophic groups, and percent cover of coral, erect macroalgae, turf algae, CCA, other invertebrates, dead coral + rock, and sand, along with density of sea urchins.

## Results

We surveyed a total of 97 nearshore locations across all four islands for algae, corals, sessile invertebrates, sea urchins, and fishes (Fig. 1, Table 1). In addition, we made 21 drop-cam deployments among all four islands to depths ranging from 78 to 1,585 m.

**Table 1:** Pitcairn Islands sampling locations by depth and habitat.

			Forereef			
Island	Island type	Lagoon	10 m	20 m	30 m	Total
Pitcairn	High island		12	12	2	26
Ducie	Atoll	3(2)*	9	9		21
Henderson	Raised atoll		13	13		26
Oeno	Atoll		12	12		24
		3(2)*	46	46	2	97

*Only 2 benthic stations were surveyed in the lagoon compared to 3 fish stations.

### Benthic Communities

#### Community Structure

Percent substrate cover varied significantly for each of the major functional groups by island (F_12, 249_ = 10.5, p<0.001), but not by depth (F_4, 81_ = 1.7, p = 0.15) or the interaction of the two terms (F_12, 249_ = 0.1, p = 0.8). A significant proportion of the variation (MANOVA, p<0.001) was explained by the four primary functional substrate groups: coral (r^2^ = 0.74), turf algae (r^2^ = 0.48), erect macroalgae (r^2^ = 0.42), and CCA (r^2^ = 0.36). Substrate cover for coral, erect macroalgae, and turf algae was not significantly different between depths (p>0.05 for all). Only CCA showed significantly lower cover at 20 m compared to the 10 m sites (p<0.05).

Coral cover was significantly greater at Ducie (56.3%±20.6 SD of the bottom) compared to the other islands, with the lowest coral cover observed at Pitcairn (5.2%±6.1 SD, Table 2). Erect macroalgae were the most prevalent benthic cover (42.1%±20.6 SD) at Pitcairn and differed significantly from the other islands, with the lowest cover at Ducie (5.8%±7.7 SD). Turf algal cover was very low at most sites except Henderson where it reached 24.0% (±17.9 SD). CCA was common at all sites and did not differ significantly among islands. CCA cover values ranged from 29.5% at Henderson to 26.2% at Ducie.

**Table 2:** Comparisons of benthic functional groups among islands.

Functional group	F	p	Multiple comparisons			
Coral	51.1	<0.001	Ducie	Oeno	Henderson	Pitcairn
			56.3 (20.6)	27.8 (10.2)	23.5 (18.0)	5.2 (6.1)
			A	B	B	C
CCA	0.2	0.910	Henderson	Oeno	Pitcairn	Ducie
			29.5 (16.8)	29.2 (24.3)	27.3 (21.0)	26.2 (16.6)
			A	A	A	A
Erect macroalgae	31.1	<0.001	Pitcairn	Oeno	Henderson	Ducie
			42.1 (20.6)	15.7 (11.7)	11.2 (13.7)	5.8 (7.7)
			A	B	BC	C
Turf algae	9.0	<0.001	Henderson	Pitcairn	Oeno	Ducie
			24.0 (17.9)	15.2 (15.6)	9.4 (1.9)	3.2 (3.6)
			A	AB	BC	C

Values are mean percent cover with one standard deviation in parentheses. Statistical results of one-way ANOVA and multiple comparisons using the Tukey-Kramer HSD test for ANOVAs. Islands with the same letter are not significantly different at α = 0.05.

#### Algae

We identified 64 macroalgal taxa (21 green algae, 12 brown algae, and 31 red algae), 51 of which are new records for these islands (Table S1). Algal species richness was greatest at Pitcairn and Henderson (42 and 31 taxa, respectively), followed by Oeno (24) and Ducie (13). Fourteen species previously reported from Pitcairn Island [44], [45] were not found in our surveys (five are likely due to taxonomic uncertainty; the other nine were likely encountered in the intertidal zone or littoral pools, environments not sampled in our surveys). Only three species of algae were common to all four islands: the brown alga *Lobophora variegata* and the encrusting corallines *Hydrolithon onkodes* and *H. gardineri*.

**Figure 2:**
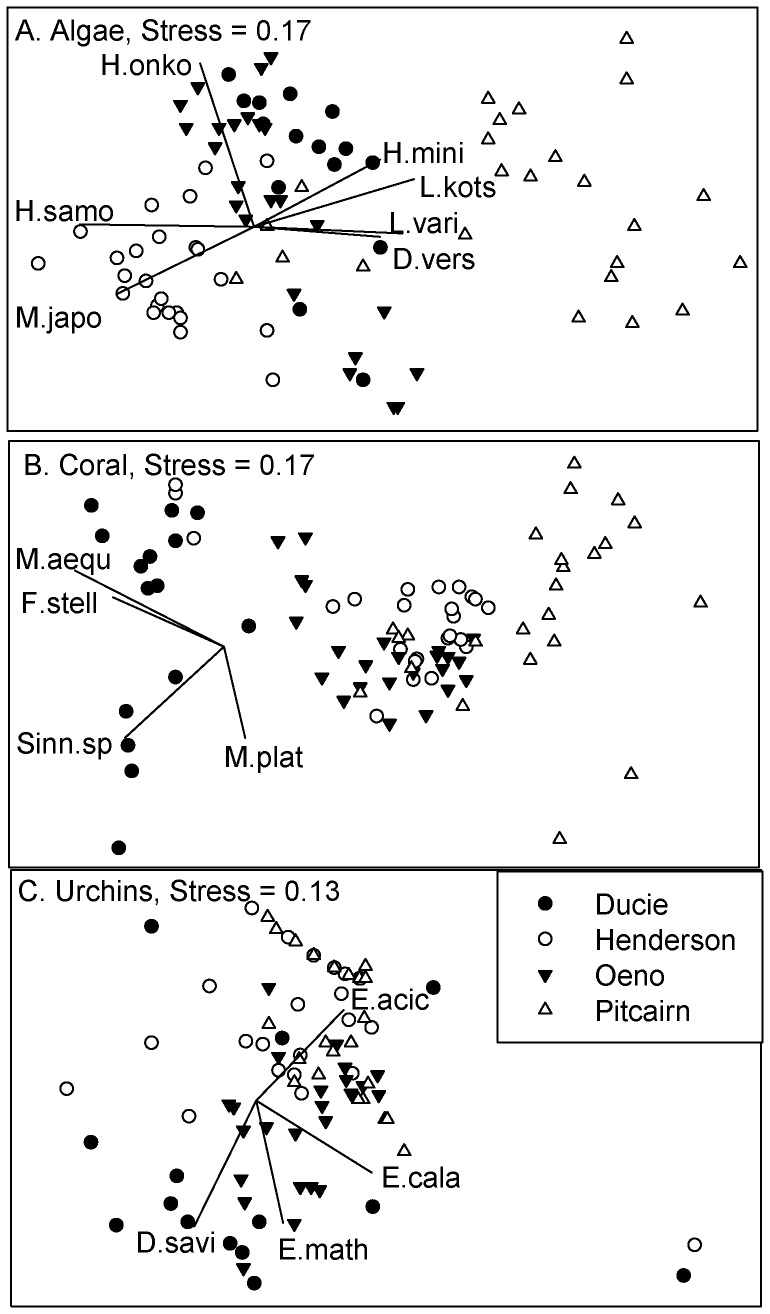
Non-metric multidimensional scaling of major benthic function groups and sampling locations among the four islands in the Pitcairn Group. A. macroalgae, B. corals, C. urchins. Vectors are the primary taxa driving the ordination (Pearson Product movement correlations ≥ 0.5). Macroalgae species codes: D.vers  =  *Dictyosphaeria versluysii*, H.onko  =  *Hydrolithon onkodes*, H. mini  =  *Halimeda minima*, H.samo  =  *Hydrolithon samoense*, M.umbr  =  *Microdictyon japonicum*, L. vari  =  erect *Lobophora variegata*. Coral species codes: F.stell  =  *Favia stelligera*, M.aequ  =  *Montipora aequituberculata*, M.plat  =  *Millepora plathyphylla*, Sinn.sp  =  *Sinularia* sp. Urchins: D.savi  =  *Diadema savignyi*, E.mata  =  *Echinometra mathaei*, E.acic  =  *Echinostrephus aciculatus*, E.cala  =  *Echinothrix calamaris*.

Algal assemblages were significantly different among islands (Global R = 0.68, Stress  = 0.15; Fig. 2A) but were indistinguishable by depth (R = 0.02). The assemblage at Pitcairn was distinct from the other three islands (all ANOSIM comparisons with Pitcairn, R>0.75). The assemblages at Henderson and Ducie were overlapping but clearly different (R = 0.72), while all other pair-wise comparisons showed even greater overlap (R>0.25 and <0.5). At Pitcairn, an erect, stipitate form of *Lobophora variegata* accounted for 26.7% of the total algal cover, followed by *Halimeda minima* (21.1%), *Lithophyllum kotschyanum* (12.5%), and an encrusting form of *Lobophora variegata* (7.1%). *Hydrolithon onkodes* (44.1%) was the most abundant species at Ducie, followed by encrusting *L. variegata* (23.0%), and *Microdictyon japonicum* (15.3%). The assemblage at Henderson consisted of *Hydrolithon samoense* (39.2%), *M. japonicum* (17.5%), *Dasya* sp. (15.1%), and encrusting *L. variegata* (14.4%). At Oeno, encrusting *L. variegata* accounted for 36.7% of the algal abundance, followed by *H. onkodes* (28.6%), and *H. samoense* (14.5%)

#### Corals

A total of 70 species of scleractinia (hard corals) were observed on quantitative benthic surveys on hard bottom substrates around the four islands (Table S2), with 23 new records for the island group. Species richness was positively correlated with geological age of the islands (r = 0.98, p = 0.02), with the oldest island Oeno (16 Mya) having the highest number of coral species (58), followed by Henderson (13 Mya: 53 species), Ducie (8 Mya: 35 species), and Pitcairn (0.8 Mya: 24 species). Nine species listed in [18] were not observed at any of the islands. Some of the species previously reported, such as *Acropora humilis*, are similar to species (e.g. *Acropora samoensis*) documented in this study, which may reflect updated taxonomy or differences in identifications.

Coral assemblages overlapped but were clearly different among islands (Global R = 0.57, Stress  = 0.16; Fig. 2B) and were indistinguishable by depth (R = 0.15). The assemblage at Ducie was distinct from the other three islands (all ANOSIM comparisons with Ducie, R>0.75). The assemblage at Pitcairn overlapped but was clearly different from Oeno and Henderson (R>0. 5 for both), while Oeno and Henderson were most similar (R = 0.26). Coral cover at Ducie was dominated by *Montipora aequituberculata* (49.9% of the total coral cover), followed by *Sinularia* sp. (14.0%), *Pavona* sp.1 (6.7%), and *Acropora valida* (6.4%). The cnidarian assemblage at Oeno consisted of *Millepora plathyphylla* (23.9%), *Pocillopora verrucosa* (15.7%), and *Porites lobata* (11.2%). At Henderson, *Pavona* sp. 1 accounted for 17.0% of total coral cover, followed closely by *P. verrucosa* (16.6%), and *M. plathyphylla* (11.6%). While coral cover at Pitcairn was lower than the other islands, the community was formed by *P. verrucosa* (21.2%), *P. lobata* (18.6%), *M. plathyphylla* (16.7%) and *Pocillopora eydouxii* (16.3%).

#### Sea Urchin Density

Sea urchins were the most abundant macro-invertebrate group encountered at all islands, and were represented by seven species (Table S3). Mean density ranged from 0 sea urchins m^−2^ for several species up to 5.4 m^−2^ sea urchins (±2.4 SD) for *Echinostrephus aciculatus* at the Pitcairn 10 m sites. At the island level, Oeno generally had the highest overall sea urchin density (7.6 m^−2^±2.7 SD) while Ducie had the lowest levels (2.4 m^−2^±2.5 SD). Urchin assemblages were similar among islands (Global R = 0.39, Stress  = 0.13; Fig. 2C) and depths (R = 0.31). Despite these overlaps, Ducie was clearly different from Pitcairn (R = 0.74) and well separated from Henderson (R = 0.52) and Oeno (R = 0.51), while Henderson and Pitcairn were indistinguishable (R = 0.16).

### Reef Fish Assemblages

#### Biodiversity

We identified a total of 205 fish species from 40 families during the expedition with 15 new records for the archipelago (Table S4). The greatest species richness was found at Henderson and Oeno (151 species for both), followed by Pitcairn (145), and Ducie (123). The majority of the fish species observed (64%) were of Indo-Pacific origin (Table 3), followed by species with Pacific-wide distributions (14%), Pitcairn regional endemics (11%), and other areas (11%). We defined Pitcairn regional endemics as species that are only found in the southeastern tropical Pacific including southern French Polynesia (e.g., Gambier, Rapa) and Easter Island. Based on density of individuals, these regional endemics accounted for 45% of the total fish assemblage (Table 4). The highest numerical abundance of regional endemics was found at Ducie (56%) with the lowest at Pitcairn (29%).

**Table 3:** Biogeographic distribution of fish species observed among the Pitcairn Islands based on species presence.

Distribution	Total	Ducie	Henderson	Oeno	Pitcairn
Anti-tropical	6 (2.9)	4 (3.3)	4 (2.6)	3 (2.0)	4 (2.8)
Circumtropical	8 (3.9)	6 (4.9)	5 (3.3)	3 (2.0)	5 (3.4)
Central Pacific	3 (1.5)	3 (2.4)	3 (2.0)	3 (2.0)	3 (2.1)
Indo-Pacific	131 (63.9)	75 (61.0)	98 (64.9)	98 (64.9)	92 (63.4)
Pacific	29 (14.1)	19 (15.4)	22 (14.6)	23 (15.2)	22 (15.2)
Pitcairn endemic^*^	1 (0.5)	1 (0.8)	1 (0.7)	1 (0.7)	1 (0.7)
Pitcairn regional endemic^#^	22 (10.7)	13 (10.6)	15 (9.9)	16 (10.6)	13 (9.0)
South Pacific	1 (0.5)	1 (0.8)	1 (0.7)	1 (0.7)	1 (0.7)
Subtropical South Pacific	4 (2.0)	1 (0.8)	2 (1.3)	3 (2.0)	4 (2.8)
Total	205	123	151	151	145

Values are numbers of species with percentages in parentheses for all four islands combined and for each island individually.

Pitcairn endemic*: only found at the Pitcairn Islands;

Pitcairn regional endemic^#^: found at Pitcairn and Easter/Salas y Gómez and/or Tuamotus/Austral Islands.

**Table 4:** Biogeographic distribution of fish species observed among the Pitcairn Islands based on and density (no. individuals m^−2^).

Distribution	Total	Ducie	Henderson	Oeno	Pitcairn
Anti-tropical	9.1	2.4	13.8	4.3	12.0
Circumtropical	0.3	0.5	0.4	0.0	0.3
Central Pacific	1.2	2.5	0.7	1.4	0.7
Indo-Pacific	22.6	20.7	13.9	29.8	46.0
Pacific	20.1	16.8	22.7	21.0	11.1
Pitcairn endemic^*^	1.7	1.8	1.1	3.3	0.0
Pitcairn regional endemic^#^	44.6	55.5	47.3	39.4	29.2
South Pacific	0.0	0.0	0.0	0.0	0.0
Subtropical South Pacific	0.4	0.0	0.1	0.8	0.7

Values are percentage of total for all four islands combined and for each island individually.

Pitcairn endemic*: only found at the Pitcairn Islands;

Pitcairn regional endemic^#^: found at Pitcairn and Easter/Salas y Gómez and/or Tuamotus/Austral Islands.

#### Fish assemblage characteristics

**Figure 3:**
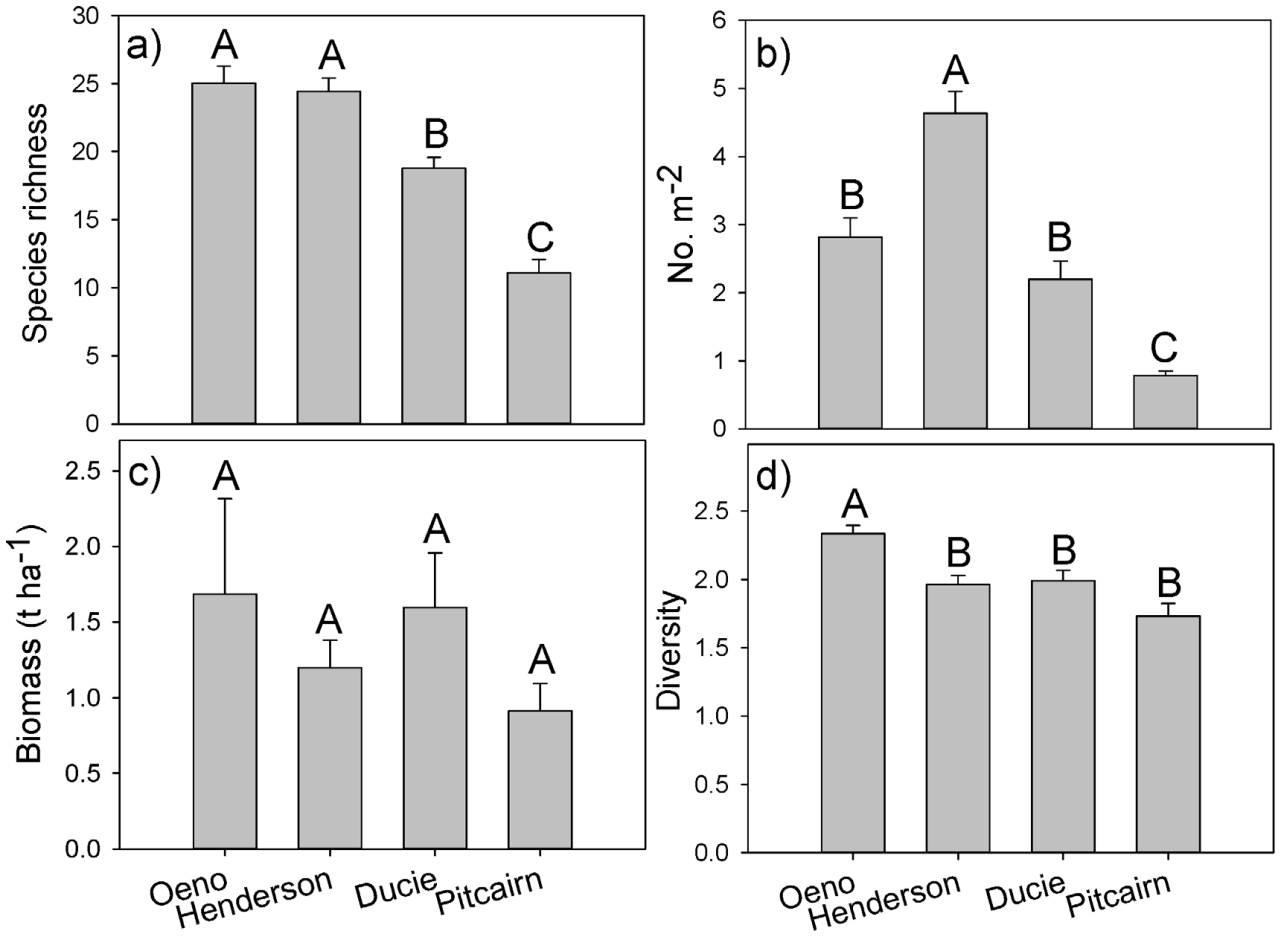
Comparison of fish assemblage characteristics among islands. A) species richness, B) numerical abundance (no. indiv. m^−2^), C) biomass (t ha^−1^), D) diversity. One-way ANOVA results for each assemblage metric are in Results. Islands with the same letter are not significantly different at α = 0.05 (Tukey's HSD tests).

Species richness differed significantly among islands (F_3, 91_ = 39.4, p<0.001) but not between depth strata (F_1, 91_ = 0.1, p = 0.8) or their interaction (F_3, 91_ = 0.7, p = 0.5). The average number of species observed on transects was highest at Oeno and Henderson and significantly different from Ducie and Pitcairn, which had the lowest richness (Fig 3a). The mean number of individuals m^−2^ differed significantly among islands (F_3, 91_ = 68.7, p<0.001) and was more than five times higher at Henderson (4.6±1.6 SD) compared to Pitcairn (0.8±0.2) with densities at Oeno and Ducie intermediate to these locations (Fig 3b). Total biomass had high within-island variance and did not differ significantly among islands (H = 6.2, p = 0.1), although biomass was 88% higher at the highest location, Oeno (1.7 t ha^−1^±3.1), compared with the lowest, Pitcairn (0.9 t ha^−1^±0.9) (Fig. 3c). Fish species diversity (H′) differed significantly among islands (F_3, 91_ = 11.2, p<0.001) with the highest diversity at Oeno (2.3±0.3) compared to similar levels among the other islands (Fig. 3d).

#### Trophic and species comparisons

**Figure 4:**
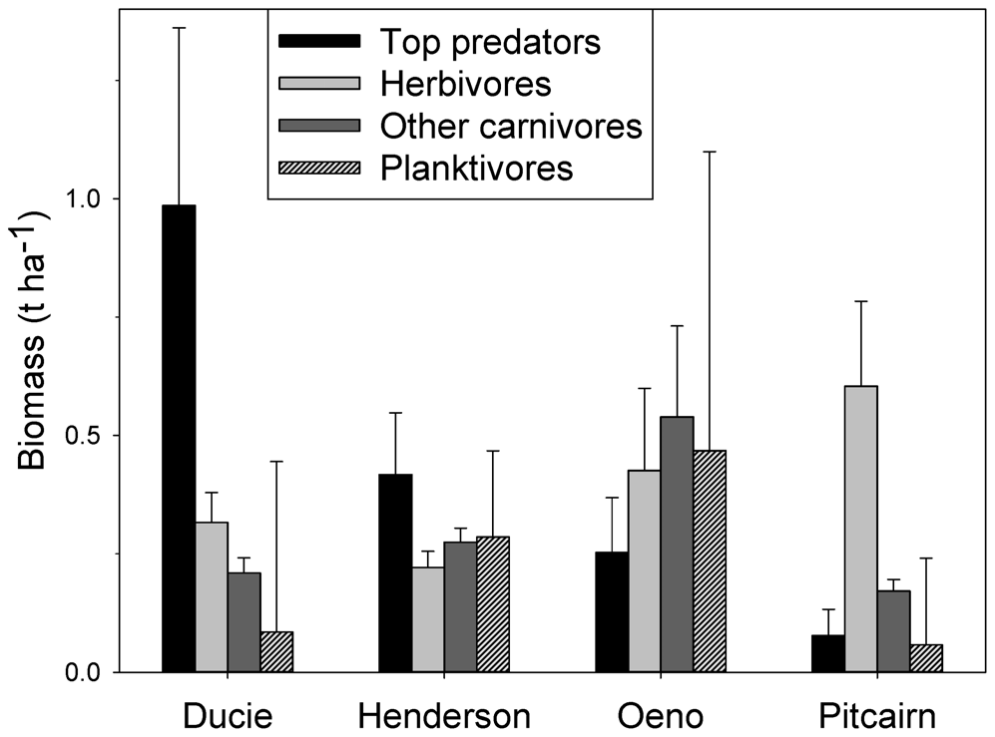
Biomass (t ha^−1^) of reef fishes by trophic group at each island of the Pitcairn islands. Error bars are standard error of the mean.

Top predators and herbivores each accounted for an average 30% of the total biomass across all islands, followed by other carnivores (23%), and planktivores (17%). However, there was a strong interaction between island and trophic group (F_9, 371_ = 2.7, p = 0.005) with top predators more abundant at Ducie (62%) and to a lesser extent at Henderson (35%). Herbivores dominated at Pitcairn (66%), while the trophic structure at Oeno was more balanced with no single dominant group (Fig. 4).

Grey reef sharks (*Carcharhinus amblyrhynchos*) comprised 46% of the top predator biomass overall, followed by whitetip reef sharks (*Triaenodon obesus* – 12%), and black trevally (*Caranx lugubris*– 10%). Biomass of herbivores consisted of chubs (*Kyphosus* spp. – 28%), unicornfish (*Naso unicornis* – 22%), and whitebar surgeonfish (*Acanthurus leucopareius* – 12%). The blacktip grouper (*Epinephelus fasciatus*) comprised 17% of the biomass of other carnivores followed by Bigeye bream (*Monotaxis grandoculis* – 9%), doublebar goatfish (*Parupeneus insularis* – 8%), and striped bream (*Gnathodentex aureolineatus* – 7%). The blotcheye soldierfish (*Myripristis berndti*) dominated the biomass of planktivores (47%), with two small (<10 cm TL) regionally endemic damselfishes (*Chromis bami* and *Chrysiptera galba*) together accounting for an additional 17% of the biomass in this trophic group.

### Structure of the coral reef community (benthos and reef fishes)

**Figure 5:**
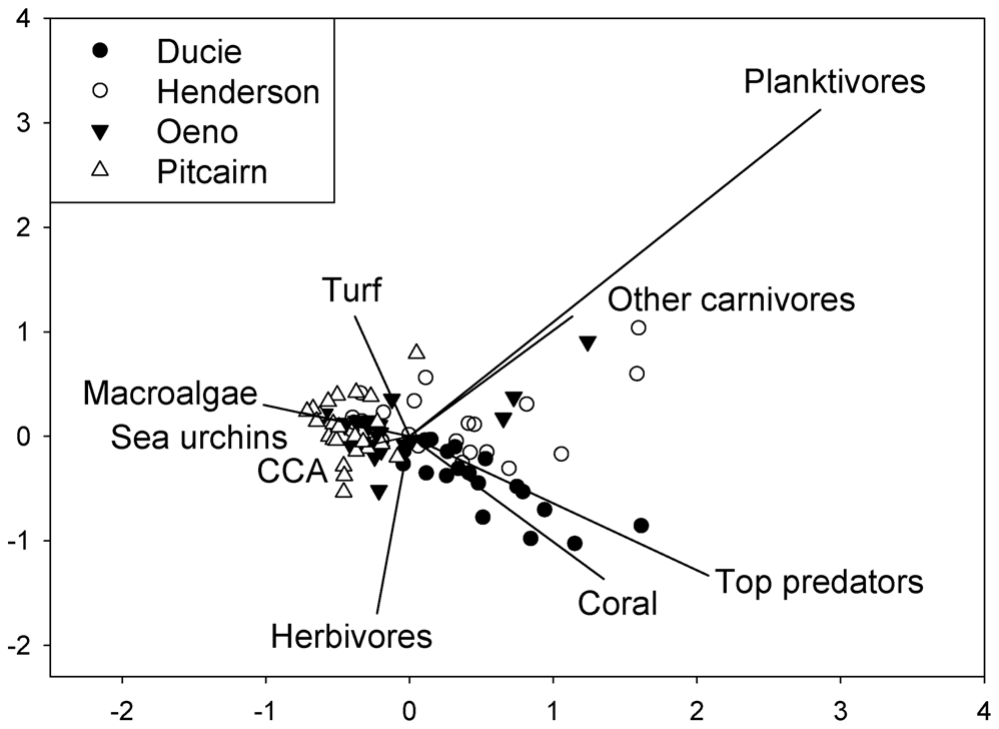
Correspondence analysis on percent cover of major benthic functional groups, abundance of sea urchins, and biomass of fish trophic groups.

The multivariate analyses showed large variability in the structure of the coral reef ecosystem (benthos and reef fishes) among sites within islands, yet obvious distinctions between islands were present (Fig. 5). Ducie was the island most clearly distinguished by the high abundance of top predators and high cover of coral. Henderson was also well separated in ordination space with other carnivores explaining most of the difference. Oeno was characterized by lower coral cover and more carnivorous fishes than Pitcairn, which was unique because of its dominance by algae. Pitcairn was the island with the highest concordance among stations. Ducie and Henderson showed the greatest variability among stations (i.e. largest spread in the plot), likely due to the large size of the islands, differences in wave exposures, and diversity of habitats.

### Deep Reefs

Fifty-seven species of deep reef fishes from 34 families were identified from drop-cam deployments, suggesting a rich deep-sea biodiversity including rare species such as the false catshark (*Pseudotriakis microdon*) (Table S5). Reef sharks, typically associated with shallow reefs, were observed as deep as 300 m, and one dogtooth tuna (*Gymnosarda unicolor*) was observed at a depth of 805 m. The ‘40 Mile Reef’, a seamount located about 75 km SE from Pitcairn, which reached to ca. 75 m of the surface, has one of the deepest well-developed coral reefs reported worldwide and consisted mostly of *Porites* cf. *deformis* and *Pocillopora* sp. Reef fishes were abundant, including predators such as the groupers *Epinephelus fasciatus* and *E. tuamotensis*, black trevally, and grey reef sharks. *Epinephelus tuamotensis* was common between 78–200 m, and it was the most common large demersal predator observed in the drop-cam footage.

We observed the presence of crustose coralline algae (CCA) at 312 m depth (and probably 382 m) (Ballesteros et al. in prep.), 44 to 114 m deeper than previously reported [46]. The drop-cam footage showed abundant CCA and probably the endolithic green alga *Ostreobium* sp. below 200 m at Ducie and Henderson, and at 312 m at Ducie. Our footage also shows a potentially deeper CCA at 382 m at Henderson. The invertebrate fauna in deeper habitats was dominated by crustaceans, mostly Mysids in the water column, and crabs (Paguridae, Parapaguridae, Galatheidae) on the bottom (Table S6). Gorgonians were the most abundant Cnidarians at depths >200 m, while two taxa of scleractinian corals (*Pocillopora* sp. and *Porites* cf. *deformis*) were observed at depths down to 100 m.

The habitats between 800–1600 m showed a lower diversity of organisms, and the presence of fishes that were not observed shallower, such as the spiny dogfish (*Squalus* sp.), the false catshark (*Pseudotriakis microdon*), snake mackerels (Gempylidae), beardfishes (Polymixidae), grenadiers (Macrouridae), duckbill eels (Nettastomatidae), and Morid cods (Moridae) (Table S5). The presence of a dogtooth tuna (*Gymnosarda unicolor*), at 805 m at Pitcairn was remarkable, and dramatically expands the known depth range of the species [47].

## Discussion

Our results indicate that the Pitcairn Islands contain healthy coral reef communities that lie at the eastern limits of the Indo-Pacific Province. Ducie was dominated by top predators and high coral cover. Although not as high as Ducie, more than 35% of the total fish biomass at Henderson consisted of top predators. The high cover of coral, particularly at Ducie and Henderson, is noteworthy because the islands are at the southern limit of coral reef distribution in the Pacific [48]. Oeno had a healthy population of carnivores, but sharks were rare. This may suggest some fishing activity at this atoll, which is the closest in the Pitcairn Group to the inhabited islands of French Polynesia. The lower coral cover in shallow waters at Pitcairn are likely influenced by runoff and sedimentation from the island, but a healthy deeper coral reef ecosystem was found further offshore. Sharks were very rarely observed at Pitcairn, as expected from an inhabited island where sharks are still fished. The structure of the food web across the island group was clearly influenced by the degree of isolation (i.e. fishing pressure), with Pitcairn and Oeno showing lower overall fish biomass and a smaller proportion of top predators. The high endemism throughout the group highlights the isolation of these islands, particularly at Ducie, which had the highest proportion of endemics and is also the most remote island in the group.

### Benthic communities

The high dissimilarity between the algal floras of the four islands is almost certainly related to the different geomorphologies, habitats, and/or isolation of these islands. Most of the marine flora in the island group is typically tropical Indo-Pacific in origin with most species also present in French Polynesia [49]–[51]. Macroalgal beds dominated by *Sargassum* spp. and an erect form of *Lobophora variegata*, which forms extensive seaweed beds at Pitcairn Island between 5 and 18 m, are also found in Lord Howe Island, Easter Island, and other southern high islands in French Polynesia [49], [52]–[53]. Ducie, Oeno, and Henderson had relatively low cover of algae, which denotes a healthy coral reef environment with high herbivore biomass. Pitcairn had more algae and less coral since it is a more nutrient-rich environment (especially in iron) because of the high island runoff. This runoff has accelerated in recent history with land modification by the local population (including building of roads and land uses) and changes in local weather patterns.

The high coral cover at Ducie (56%) was exceptional considering this island is the southernmost atoll in the world and near the easternmost limit of coral reef distribution in the Pacific. The coral cover was comparable to several other significant high latitude reefs [54]–[55]. Consequently, Ducie should be considered a high priority conservation site given its current lack of local human impacts and the potential to be more resilient to climate change [56]. Oeno and Henderson also had significant coral cover (28% and 24%, respectively) despite being at the southern limit of coral distribution. At Pitcairn we found a deep coral reef (developing below 35 m depth) that had not been recorded previously, with a remarkable 26% of the bottom covered by live coral. The coral cover of mesophotic reefs at Pitcairn is higher than those observed at similar latitudes in the Northwestern Hawaiian Islands (17%, [57]) and consisted of a wide range of species. The extreme water clarity surrounding the Pitcairn Islands (measured up to 75 m at Ducie) allows for coral growth at depths greater than expected for most Pacific reefs [58]. This deeper available habitat may help build resilience into ecosystems from potential climate change impacts [59]. In addition, Pitcairn is located near the center of the South Pacific Circulation Gyre, and climate change predictions suggest that this region will show less dramatic changes in SST, carbonate, and pH than most other regions around the globe [60].

The positive correlation between coral species richness and geological age of the islands, while not surprising, highlights some intriguing biogeographical patterns. The older islands have experienced greater reef development and coral proliferation than the younger islands due to the longer colonization time. This pattern has been documented with many taxonomic groups throughout the Pacific [61] and is an important component of island biogeographic theory [62]. Holocene reef growth over the past 11,000 years would have been relatively similar among the four islands. Pitcairn likely did not benefit from prior species introductions during the Pleistocene when it was still tectonically active [63] and when Henderson was experiencing high coral reef growth [64]. The older islands were geographically closer to French Polynesia, which has higher coral species richness [65] and therefore it is reasonable to assume that coral species richness would decrease with increasing remoteness. The exception to this hypothesis is Pitcairn, which is closer to the Gambier Islands than Ducie and Henderson, yet had the lowest coral species richness. While it is possible that the uniqueness of Pitcairn (i.e. only high island coupled with anthropogenic impacts) may have contributed to the lower species richness, a more likely explanation is that Pitcairn is the only emergent island along the more southerly and geological ‘hotspot’ region [18]. In comparison, the other three islands lie along the more northerly geological ‘hotspot’ region that is parallel to Pitcairn and closer in proximity to the Gambier island group. This northern underwater ridge, with a more extensive shallow water habitat [18] than the isolated southerly ridgeline with Pitcairn, might have enhanced colonizing fauna and flora by acting as stepping stones from the Gambier island group [61]. Even though this colonization pattern is counter to the direction of the prevailing winds and currents coming from the east in the central South Pacific [21], the species distribution patterns suggests that species moved from west to east and from older islands to younger islands, inferring that the current patterns must have been reversed on occasion.

### Fishes

Total reef fish biomass for all islands combined was relatively low (ca. 1.4 t ha^−1^) compared to other uninhabited islands situated further north in the central Pacific such as the Line Islands, where unfished fish biomass can exceed 5 t ha^−1^ in some places [36], [66]. The relatively low biomass at the Pitcairn Islands may be due to the extremely low productivity of the waters of the Pitcairn EEZ, compared to the waters in much of the Pacific Ocean [67], [68]. The low productivity results in low plankton abundance, which results in extremely clear waters [69]. Nevertheless, the fish biomass found at the Pitcairn Islands is larger than most fished sites in the Indo-Pacific and the Caribbean [36], [70]–[72].

Total fish biomass in the tropical Pacific is determined by the background productivity of the oceanic waters [73]–[74] and possibly the level of species diversity. However, the health of the fish assemblages is determined by the degree of fishing: lower fishing pressure results in a larger proportion of the fish biomass that is accounted for by predators since fishers typically target the largest individuals in a population [75]–[76]. The 62% top predator biomass at Ducie is one of the largest recorded [23], [36], [77], which is particularly notable because it was not completely driven by a few large sharks, but rather by a large number of top predators including groupers and snappers.

### The deep waters of the Pitcairn EEZ

We conducted the first survey of deep-sea life in the Pitcairn Islands EEZ (notwithstanding previous fishing surveys of the relatively shallow seamount ‘40 Mile Reef’). We identified 57 species of fishes in only 21 drop-cam deployments. Taking into account that the average size of the area filmed by the drop-cam is only 3 m^2^, the diversity of fish found on the deep habitats of the Pitcairn Islands is notable. By comparison, similar drop-cam deployments around Easter and Salas y Gomez islands, 2000 km further to the east along the Nazca Ridge, yielded only 26 fish species [53].

The abundance of groupers and sharks at depths between 100–300 m also indicates the intactness of these deep fish populations, especially at ‘40 Mile Reef’, which harbors a high fish biomass and is one of the deepest well-developed coral reef communities currently known [78]. Seamounts worldwide are being trawled, depleted, and abandoned, and their recovery seems unlikely within our lifetime, or not at all, because many target species are long-lived, mature late, and have a small reproductive output [79]–[81]. The Pitcairn Islands seamounts appear to be relatively intact, and therefore have high global conservation value.

We found eight probable new species of reef fishes on our deep camera surveys, mostly between 100–300 m, which suggests that more extensive surveys will probably yield many more species new to science. Determining how many of these new species are endemic to the Pitcairn Islands or are regional endemics will require additional sampling and collections. In addition, the extreme water clarity allows marine plants at the Pitcairn Islands to live deeper than in any other reported location on earth. The previous depth record for benthic algae was CCA observed at 268 m in the Bahamas [82]. In summary, our findings clearly show the unique biodiversity in the deep habitats of the Pitcairn Islands EEZ and the need to explore these deeper habitats elsewhere.

## Conclusions

Because of the nearly pristine and unique nature of most marine ecosystems of the Pitcairn Islands, its EEZ has a unique global value that is irreplaceable. There are only a handful of areas in the EEZs of the world that remain pristine, occupying probably less than 5% of the ocean [83]. These places allow us to envision what the ocean was like before heavy human impacts, to understand what we have lost in other places because of human impacts, and most importantly, to set proper conservation and management goals for our oceans [23]–[24].

Pitcairn islands and the surrounding EEZ are currently being considered for protection in what would be the largest marine reserve in the world, containing approximately 836,000 km^2^. In September 2012, the Pitcairn community unanimously agreed to support the creation of a marine reserve, and in January 2013 a joint proposal was submitted to the UK Government for consideration. If protection of this area proceeds, scientific research and monitoring will be established. This study, as the first to quantitatively assess the community structure of the organisms inhabiting the coral reefs on the Pitcairn islands, will provide a valuable baseline by which future changes in ecosystem components can be measured.

## Supporting Information

Table S1 **List of algal species observed during expedition to Pitcairn Island group.** X =  Previous documented and observed during our surveys. X =  observed during our surveys but not previously documented. O =  observed in previous surveys but not during our surveys.
(DOCX)

Table S2 **List of coral species observed during expedition to Pitcairn Island group.** X =  Previous documented and observed during our surveys. **X** =  observed during our surveys but not previously documented. O =  observed in previous surveys but not during our surveys.
(DOCX)

Table S3 **Sea urchin density (mean no. individuals m^−2^) and standard deviation (in parentheses) within each depth (m) stratum at each island.** ***N*** **=  number of samples (sites).**
(DOCX)

Table S4 **Fish species list from Pitcairn Islands. Order is phylogenetic.** X =  Previous documented and observed during our surveys. **X** =  observed during our surveys but not previously documented. O =  observed in previous surveys (Irving et al. 1995, Randall 1999) but not observed during this survey.
(DOCX)

Table S5 **Fishes observed in deep habitats of the Pitcairn islands, using National Geographic's Drop-Cams.**
(DOCX)

Table S6 **Invertebrates observed in deep habitats of the Pitcairn islands, using National Geographic's Drop-Cams.**
(DOCX)
