# Supplementary material for: The Real Bounty: Marine Biodiversity in the Pitcairn Islands
Source: PLoS One. 2014 Jun 25;9(6):e100142. doi: 10.1371/journal.pone.0100142 (PMC4070931; doi:10.1371/journal.pone.0100142)
Supplement: Table S1 — List of algal species observed during expedition to Pitcairn Island group. X = Previous documented and observed during our surveys. X = observed during our surveys but not previously documented. O = observed in previous surveys but not during our surveys. (DOCX) [file pone.0100142.s001.docx]

Table S1. List of algal species observed during expedition to Pitcairn Island group. X = Previous documented and observed during our surveys. **X** = observed during our surveys but not previously documented. O = observed in previous surveys but not during our surveys.

| Species | Ducie | Henderson | Oeno | Pitcairn | New records for all islands | | Notes | |
| --- | --- | --- | --- | --- | --- | --- | --- | --- |
| GREEN ALGAE |  |  |  |  |  |  | |  |
| *Avrainvillea erecta* |  | **X** | **X** | **X** | YES |  | |  |
| *Bryopsis pennata* |  |  |  | O |  | Reported from Pitcairn by Tsuda (1976) | |  |
| *Caulerpa cupressoides* |  | **X** |  |  | YES |  | |  |
| *Caulerpa cupressoides urvilleana* |  | **X** | **X** |  | YES |  | |  |
| *Caulerpa nummularia* |  |  |  | **X** | YES |  | |  |
| *Caulerpa pickeringii* |  | X | X | O |  | Reported from Oeno and Henderson by N'Yeurt & Payri (2007) and from Pitcairn by Tsuda (1976) | |  |
| *Caulerpa racemosa* |  |  |  | X |  | Reported from Pitcairn by Tsuda (1976) | |  |
| *Caulerpa* cf. *seuratii* |  | **X** |  |  | YES |  | |  |
| *Cladophora coelothrix* | O |  |  | O |  | Reported from Pitcairn by Tsuda (1976) and from Ducie by Reher and Randall (1975) | |  |
| *Cladophora herpestica* |  |  |  | **X** | YES |  | |  |
| *Cladophora* sp. 1 | **X** |  |  |  | YES |  | |  |
| *Cladophora* sp. 2 (turf) |  | **X** |  |  | YES |  | |  |
| *Cladophora* sp. 3 (overhangs) |  | **X** |  |  | YES |  | |  |
| *Cladophoropsis gracillima* |  |  |  | O |  | Reported from Pitcairn by Tsuda (1976) | |  |
| *Codium geppiorum* |  |  | **X** |  | YES |  | |  |
| *Codium* sp. |  | **X** |  |  | YES |  | |  |
| *Dictyosphaeria cavernosa* |  |  |  | O |  | Reported from Pitcairn by Tsuda (1976). Misidentified with *Dictyosphaeria versluysii* | |  |
| *Dictyosphaeria versluysii* |  |  | **X** | **X** | YES |  | |  |
| *Halimeda discoidea* |  | **X** |  |  | YES |  | |  |

Table S1. Continued.

| Species | Ducie | Henderson | Oeno | Pitcairn | New records for all islands | | Notes | |
| --- | --- | --- | --- | --- | --- | --- | --- | --- |
| *Halimeda incrassata* |  | O |  | O |  | Reported from Pitcairn and Henderson by Tsuda (1976). Probably misidentified with other *Halimeda* species | |  |
| *Halimeda minima* |  |  |  | **X** | YES |  | |  |
| *Microdictyon boergesenii* | O | O |  |  |  | Reported from Ducie and Henderson by Tsuda (1976). Probably misidentified with *Microdictyon umbilicatum* | |  |
| *Microdictyon japonicum* | **X** | **X** | **X** |  | YES |  | |  |
| *Neomeris* sp. |  | **X** | **X** |  | YES |  | |  |
| *Valonia aegagropila* | **X** |  |  |  | YES |  | |  |
| *Valonia macrophysa* |  |  |  | **X** | YES |  | |  |
| *Verdigellas* cf*. peltata* |  | **X** | **X** |  | YES |  | |  |
|  |  |  |  |  |  |  | |  |
| BROWN ALGAE |  |  |  |  |  |  | |  |
| *Asteronema breviarticulatum* | **X** |  |  | O |  | Reported from Pitcairn by Tsuda (1976) | |  |
| *Canistrocarpus* cf. *magneanus* |  |  |  | **X** | YES |  | |  |
| *Dictyota acutiloba* |  |  |  | O |  | Reported from Pitcairn by Tsuda (1976) | |  |
| *Dictyota bartayresiana* |  |  | **X** | **X** | YES |  | |  |
| *Dictyota ceylanica* |  |  | **X** |  | YES |  | |  |
| *Dictyota* cf*. friabilis* |  | **X** |  |  | YES |  | |  |
| *Dictyota humifusa* |  |  |  | **X** | YES |  | |  |
| *Hydroclathrus clathratus* |  |  | **X** |  | YES |  | |  |
| *Lobophora* *variegata* (stiped) |  |  |  | **X** |  |  | |  |
| *Lobophora variegata* (encrusting) | **X** | X | **X** | X |  | Reported from Pitcairn and Henderson by Tsuda (1976) | |  |

Table S1. Continued.

| Species | Ducie | Henderson | Oeno | Pitcairn | New records for all islands | | Notes | |
| --- | --- | --- | --- | --- | --- | --- | --- | --- |
| *Padina boryana* |  |  |  | **X** | YES |  | |  |
| *Sargassum aquifolium* |  |  |  | O |  | Reported from Pitcairn by Mattio et al. (2008) (as *Sargassum echinocarpum*) | |  |
| *Sargassum coriifolium* |  |  |  | O |  | Reported from Pitcairn by Tsuda (1976). Should be a misidentification with other *Sargassum* species. | |  |
| *Sargassum obtusifolium* |  |  | **X** | X |  | Reported from Pitcairn by Mattio et al. (2008) | |  |
| *Sphacelaria* sp. |  |  |  | **X** | YES |  | |  |
| *Sphacelaria tribuloides* |  |  |  | O |  | Reported from Pitcairn by Tsuda (1976) | |  |
| *Stypopodium australasicum* |  |  | **X** | X |  | Reported from Pitcairn by N'Yeurt & Payri (2006) | |  |
| *Stypopodium zonale* |  | O |  | O |  | Reported from Pitcairn by Tsuda (1976) and from Henderson by Paulay (1989). Should be a misidentification with *S. australasicum* | |  |
|  |  |  |  |  |  |  | |  |
| RED ALGAE |  |  |  |  |  |  | |  |
| *Acrosymphyton* sp. |  | **X** |  |  | YES |  | |  |
| *Actinotrichia* sp. |  | **X** |  |  | YES |  | |  |
| *Amphiroa fragilissima* |  | **X** |  |  | YES |  | |  |
| *Botryocladia skottsbergii* |  |  |  | O |  | Reported from Pitcairn by Tsuda (1976) | |  |
| *Centroceras clavulatum* |  |  |  | O |  | Reported from Pitcairn by Tsuda (1976) | |  |
| Ceramiales (unidentified) |  |  | **X** |  | YES |  | |  |
| *Ceratodictyon intricatum* |  |  |  | X |  | Reported from Pitcairn by Tsuda (1976) and N'Yeurt & Payri (2010) (as *Gelidiopsis intricata*) | |  |

Table S1. Continued.

| Species | Ducie | Henderson | Oeno | Pitcairn | New records for all islands | | Notes | |
| --- | --- | --- | --- | --- | --- | --- | --- | --- |
| *Chondria intertexta* |  |  |  | O |  | Reported from Pitcairn by Tsuda (1976) as *Chondria intricata* | |  |
| *Dasya* cf. *anastomosans* |  |  | **X** |  | YES |  | |  |
| *Dasya* sp. |  | **X** | **X** | **X** | YES |  | |  |
| *Ganonema papenfussii*? |  |  | **X** |  | YES |  | |  |
| *Gibsmithia hawaiiensis* |  |  | **X** |  | YES |  | |  |
| *Haloplegma duperreyi* |  |  |  | **X** | YES |  | |  |
| *Hydrolithon farinosum* |  |  |  | **X** | YES |  | |  |
| *Hydrolithon gardineri* | X | **X** | **X** | **X** |  | Reported from Ducie by Rehder & Randall (1975) | |  |
| *Hydrolithon onkodes* | X | **X** | **X** | **X** |  | Reported from Ducie by Rehder & Randall (1975) | |  |
| *Hydrolithon samoense* | **X** | **X** | **X** |  | YES |  | |  |
| *Hypnea* sp. |  |  |  | **X** | YES |  | |  |
| *Hypnea pannosa* |  | **X** | **X** |  | YES |  | |  |
| *Jania pumila* |  |  |  | **X** | YES |  | |  |
| *Jania rosea* |  |  |  | O |  | Reported from Pitcairn by Tsuda (1976) as *Corallina cuvieri* | |  |
| *Jania subulata* |  |  |  | X |  | Reported from Pitcairn by N'Yeurt & Payri (2010) (as *Haliptilon subulatum*) | |  |
| *Jania* sp. |  | **X** |  |  | YES |  | |  |
| *Liagora* sp. |  |  | **X** |  | YES |  | |  |
| *Liagora ceranoides* |  | **X** |  |  | YES |  | |  |
| *Lithophyllum flavescens* | **X** | **X** |  | **X** | YES |  | |  |
| *Lithophyllum kotschyanum* |  |  |  | **X** | YES |  | |  |

Table S1. Continued.

| Species | Ducie | Henderson | Oeno | Pitcairn | New records for all islands | | Notes | |
| --- | --- | --- | --- | --- | --- | --- | --- | --- |
| *Lomentaria corallicola*? |  |  |  | **X** | YES |  | |  |
| *Neogoniolithon frutescens* | **X** | **X** |  |  | YES |  | |  |
| *Peyssonnelia boergesenii* |  | **X** |  |  | YES |  | |  |
| *Peyssonnelia conchicola* | **X** | **X** |  | **X** | YES |  | |  |
| *Pneophyllum conicum* |  |  | **X** | **X** | YES |  | |  |
| Rhodomelaceae (unidentified) | | **X** | **X** |  | YES |  | |  |
| *Sporolithon episoredion* |  | **X** |  |  | YES |  | |  |
| *Sporolithon ptychoides* |  | **X** |  |  | YES |  | |  |
| TOTAL NUMBER OF SPECIES | 13 | 31 | 24 | 42 | 51 |  | |  |

References:

Guiry, M.D. & Guiry, G.M. 2014. AlgaeBase. World-wide electronic publication, National University of Ireland, Galway. http://www.algaebase.org; searched on 07 January 2014.

Mattio L, Payri CE, Stiger-Pouvreau V. 2008. Taxonomic Revision of *Sargassum* (Fucales, Phaeophyceae) From French Polynesia Based on Morphological and Molecular Analyses. Journal of Phycology 44:1541-1555.

N'Yeurt ADR, Payri CE. 2006. Marine algal flora of French Polynesia I. Phaeophyceae (Ochrophyta, brown algae). Cryptogamie Algologie 27:111-152.

N'Yeurt ADR, Payri CE. 2007. Marine algal flora of French Polynesia II. Chlorophyceae (green algae). Cryptogamie Algologie 28:3-88.

N'Yeurt ADR, Payri CE. 2010. Marine algal flora of French Polynesia III. Rhodophyta, with additions to the Phaeophyceae and Chlorophyta. Cryptogamie Algologie 31:3-205.

Rehder HA, Randall JE. 1975. Ducie Atoll: its history, physiography and biota. Atoll Research Bulletin 183:1-40.

Tsuda RT. 1976. Some marine benthic algae from Pitcairn Island. Revue Algologique (NS) 11:325-331.
