# Supplementary material for: The Real Bounty: Marine Biodiversity in the Pitcairn Islands
Source: PLoS One. 2014 Jun 25;9(6):e100142. doi: 10.1371/journal.pone.0100142 (PMC4070931; doi:10.1371/journal.pone.0100142)
Supplement: Table S2 — List of coral species observed during expedition to Pitcairn Island group. X = Previous documented and observed during our surveys. X = observed during our surveys but not previously documented. O = observed in previous surveys but not during our surveys. (DOCX) [file pone.0100142.s002.docx]

Table S2. List of coral species observed during expedition to Pitcairn Island group. X = Previous documented and observed during our surveys. **X** = observed during our surveys but not previously documented. O = observed in previous surveys but not during our surveys.

| Species | Ducie | Henderson | Oeno | Pitcairn | New record for  all islands | Notes |
| --- | --- | --- | --- | --- | --- | --- |
|  |  |  |  |  |  |  |
| *Acropora acuminata* | X |  | X | O |  |  |
| *Acropora austera* | **X** |  | **X** |  | Yes |  |
| *Acropora cf. elizabethensis* |  |  | **X** |  | Yes |  |
| *Acropora cf. listeri* | **X** | **X** | **X** |  |  |  |
| *Acropora cf. solitaryensis* | X |  | **X** | **X** |  |  |
| *Acropora cf. vaughani* | **X** | **X** |  |  | Yes |  |
| *Acropora cytherea* |  |  | O |  |  | Reported from Oeno by Irving and Dawson (2012) |
| *Acropora digitifera* | **X** | X | **X** | **X** |  |  |
| *Acropora gemmifera* |  |  | **X** |  |  |  |
| *Acropora glauca* | **X** | X | **X** |  |  |  |
| *Acropora globiceps* |  | **X** | **X** |  |  |  |
| *Acropora humilis* | O | O | O |  |  | Reported from Ducie, Henderson, and Oeno by Irving and Dawson (2012). |
| *Acropora hyacinthus* | **X** |  | **X** |  |  |  |
| *Acropora latistella* | X | **X** |  |  |  |  |
| *Acropora lutkeni* | X | **X** | **X** | **X** |  |  |
| *Acropora microclados* | X | **X** | **X** | **X** |  |  |
| *Acropora monticulosa* |  | **X** | **X** |  | Yes |  |
| *Acropora nasuta* | X | X | **X** |  |  |  |
| *Acropora palifera* |  |  | **X** |  | Yes |  |
| *Acropora retusa* | **X** | **X** | **X** |  | Yes |  |

Table S2. Continued.

| Species | Ducie | Henderson | Oeno | Pitcairn | New record for  all islands | Notes |
| --- | --- | --- | --- | --- | --- | --- |
| *Acropora samoensis* | **X** | **X** | **X** | **X** |  |  |
| *Acropora secale* | **X** |  | X |  |  |  |
| *Acropora valida* | X | **X** | **X** |  |  |  |
| *Alveopora verrilliana* |  | **X** | **X** |  | Yes |  |
| *Astreapora cf. moretonensis* |  | O |  |  |  | Reported from Henderson by Irving and Dawson (2012) |
| *Astreopora cf. scabra* |  | **X** |  |  | Yes |  |
| *Astreopora myriophthalma* | X | **X** | **X** | X |  |  |
| *Astreopora randalli* |  | **X** | **X** |  | Yes |  |
| *Caulastrea cf. furcata* |  |  |  | O |  | Reported from Pitcairn by Irving and Dawson (2012) |
| *Coscinararea columna* |  | **X** | **X** |  | Yes |  |
| *Cycloseris vaughani* |  |  |  | O |  | Reported from Pitcairn by Irving and Dawson (2012) |
| *Cyphastrea chalcidicum* |  | **X** | **X** |  | Yes |  |
| *Cyphastrea cf. serailia* | X | X | X |  |  |  |
| *Favia matthaii* | **X** | X |  | X |  |  |
| *Favia rotumana* |  | X | **X** |  |  |  |
| *Favia speciosa* |  |  | **X** | **X** | Yes |  |
| *Favia stelligera* | X | **X** | **X** |  |  |  |
| *Fungia scutaria* | X | **X** | **X** |  |  |  |
| *Goniastrea retiformis* |  |  |  | **X** | Yes |  |
| *Goniastrea australiensis* |  | X |  | O |  |  |
| *Leptastrea pruinosa* |  | **X** | **X** | **X** | Yes |  |
| *Leptastrea purpurea* |  | X | X | X |  |  |
| *Leptastrea transversa* |  |  | O |  |  | Reported from Henderson and Oeno by Irving and Dawson (2012) |
| *Leptoria phrygia* |  |  | **X** |  | Yes |  |

Table S2. Continues.

| Species | Ducie | Henderson | Oeno | Pitcairn | New record for  all islands | Notes |
| --- | --- | --- | --- | --- | --- | --- |
| *Leptoseris cf. hawaiiensis* | **X** | **X** |  | O |  | Reported from Pitcairn by Irving and Dawson (2012) |
| *Leptoseris incrustans* | O | X | **X** |  |  |  |
| *Leptoseris mycetoseroides* |  | **X** |  |  | Yes |  |
| *Lobophyllia hemprichii* |  |  | **X** |  | Yes |  |
| *Montastrea curta* | X | X | X |  |  |  |
| *Montipora aequituberculata* | X | X | **X** |  |  |  |
| *Montipora caliculata* | X | X | **X** | **X** |  |  |
| *Montipora capitata* |  | O |  |  |  | Reported from Henderson by Irving and Dawson (2012) |
| *Montipora foveolata* | X | X | X | **X** |  |  |
| *Montipora grisea* |  |  | X |  |  |  |
| *Montipora incrassata* | X | **X** | X |  |  |  |
| *Montipora nodosa* |  |  | **X** |  | Yes |  |
| *Montipora tuberculosa* |  |  | X |  |  |  |
| *Montipora verrucosa* | O | **X** | X |  |  |  |
| *Pavona clavus* |  |  |  | **X** | Yes |  |
| *Pavona maldivensis* |  | X | **X** |  |  |  |
| *Pavona sp. 1* | X | X | **X** |  |  | Paulay (1989) states that it is intermediate in form between P. maldivensis and P. clavus/P. minuta. |
| *Pavona varians* |  | X | X |  |  |  |
| *Plesiastrea versipora* | O | O | O | O |  | Reported from Ducie, Henderson, and Oeno by Irving and Dawson (2012) |
| *Pocillopora damicornis* |  | **X** | **X** | X |  |  |
| *Pocillopora eydouxi* | X | X | **X** | **X** |  |  |
| *Pocillopora meandrina* | **X** | **X** | **X** | **X** |  |  |

Table S2. Continued.

| Species | | Ducie | Henderson | Oeno | | Pitcairn | | New record for  all islands | | Notes |
| --- | --- | --- | --- | --- | --- | --- | --- | --- | --- | --- |
| *Pocillopora verrucosa* | | **X** | **X** | **X** | | **X** | |  | | Listed as possible new species by Paulay (1989) |
| *Pocillopora woodjonesi* | |  | **X** |  | | **X** | |  | |  |
| *Porites aff.annae* | | X | X | X | | X | |  | |  |
| *Porites australiensis* | | O | X | **X** | | **X** | |  | |  |
| *Porites cf. arnaudi* | |  | **X** | **X** | |  | | Yes | |  |
| *Porites deformis* | |  |  |  | |  | | Yes | | Observed with the Drop-Cam at 40-mile Reef |
| *Porites lobata* | | X | X | **X** | | X | |  | |  |
| *Porites lutea* | |  |  | **X** | |  | | Yes | |  |
| *Porites cf. profundus* | |  | **X** |  | |  | | Yes | |  |
| *Psammocora haimeana* | |  | X | X | | X | |  | |  |
| *Psammocora nierstraszi* | | **X** | **X** | **X** | |  | | Yes | |  |
| *Psammocora obtusangula* | | X | X | **X** | |  | |  | |  |
| *Scolymia cf. vitiensis* | |  | X |  | | X | |  | |  |
| *Stylocoeniella guentheri* | |  | O |  | | O | |  | | Reported from Henderson and Pitcairn by Irving and Dawson (2012) |
| Total number of species | | 40 | 58 | 62 | | 31 | | 23 | |  |
| Total species observed: 79 - 9 reported but not observed = 70 | | | |  | |  | |  |  |  |

References

Irving RA, Dawson TP (2012) The marine environment of the Pitcairn Islands. A report to Global Ocean Legacy, a project of the Pew Environment Group. Dundee: Dundee University Press. 106 p.

Paulay G (1989) Marine invertebrates of the Pitcairn Islands: species composition and biogeography of corals, molluscs and echinoderms. Atoll Research Bulletin 326: 1-28.
