# Supplementary material for: The Real Bounty: Marine Biodiversity in the Pitcairn Islands
Source: PLoS One. 2014 Jun 25;9(6):e100142. doi: 10.1371/journal.pone.0100142 (PMC4070931; doi:10.1371/journal.pone.0100142)
Supplement: Table S3 — Sea urchin density (mean no. individuals m−2) and standard deviation (in parentheses) within each depth (m) stratum at each island. N = number of samples (sites). (DOCX) [file pone.0100142.s003.docx]

Table S3. Sea urchin density (mean no. individuals m^-2^) and standard deviation (in parentheses) within each depth (m) stratum at each island. *N* = number of samples (sites).

| Island | Ducie | | Henderson | | Oeno | | Pitcairn | | |
| --- | --- | --- | --- | --- | --- | --- | --- | --- | --- |
| Depth (m) | 10 | 20 | 10 | 20 | 10 | 20 | 10 | 20 | 30 |
| *N* | 11 | 9 | 13 | 13 | 12 | 12 | 12 | 12 | 2 |
|  |  |  |  |  |  |  |  |  |  |
| *Diadema savignyi* | 0.61 (1.27) | 0.98 (1.27) | 0.16 (0.20) | 0.21 (0.27) | 0.32 (0.45) | 2.71 (2.02) | 0.00  (-) | 0.07 (0.23) | 2.53 (2.83) |
| *Echinometra mathaei* | 0.99 (1.46) | 0.62 (1.31) | 0.37 (0.46) | 0.06 (0.16) | 3.09 (2.06) | 1.67 (1.64) | 3.07 (3.18) | 0.20 (0.36) | 0.13 (0.19) |
| *Echinometra oblonga* | 0.07 (0.24) | 0.00  (-) | 0.04 (0.10) | 0.00  (-) | 0.23 (0.32) | 0.00  (-) | 0.36 (0.63) | 0.02 (0.08) | 0.00  (-) |
| *Echinostrephus aciculatus* | 0.39 (0.62) | 0.00  (-) | 4.41 (2.40) | 1.60 (2.18) | 4.91 (2.18) | 1.67 (1.13) | 5.40 (2.36) | 3.91 (2.24) | 1.07 (1.51) |
| *Echinothrix calamaris* | 0.05 (0.11) | 0.03 (0.09) | 0.00  (-) | 0.04 (0.15) | 0.00  (-) | 0.04 (0.10) | 0.02 (0.08) | 0.00  (-) | 0.00  (-) |
| *Echinothrix diadema* | 0.00  (-) | 0.00  (-) | 0.02 (0.07) | 0.00  (-) | 0.07 (0.17) | 0.00  (-) | 0.00  (-) | 0.04 (0.10) | 0.00  (-) |
| *Heterocentrotus mammillatus* | 0.44 (0.54) | 0.00  (-) | 0.04 (0.10) | 0.00  (-) | 0.43 (0.81) | 0.02 (0.08) | 0.02 (0.08) | 0.00  (-) | 0.00  (-) |
|  |  |  |  |  |  |  |  |  |  |
