# Supplementary material for: The Real Bounty: Marine Biodiversity in the Pitcairn Islands
Source: PLoS One. 2014 Jun 25;9(6):e100142. doi: 10.1371/journal.pone.0100142 (PMC4070931; doi:10.1371/journal.pone.0100142)
Supplement: Table S4 — Fish species list from Pitcairn Islands. Order is phylogenetic. X = Previous documented and observed during our surveys. X = observed during our surveys but not previously documented. O = observed in previous surveys (Irving et al. 1995, Randall 1999) but not observed during this survey. (DOCX) [file pone.0100142.s004.docx]

Table S4. Fish species list from Pitcairn Islands. Order is phylogenetic. X = Previous documented and observed during our surveys. **X** = observed during our surveys but not previously documented. O = observed in previous surveys (Irving et al. 1995, Randall 1999) but not observed during this survey.

| Family | Taxon | Ducie | Henderson | Oeno | Pitcairn | New record for all islands |
| --- | --- | --- | --- | --- | --- | --- |
| Carcharhinidae | *Carcharhinus amblyrhynchos* | X | X | X | X |  |
|  | *Carcharhinus falciformis* | **X** |  |  |  | **X** |
|  | *Triaenodon obesus* | X | X | X | X |  |
| Myliobatidae | *Aetobatis narinari* | **X** |  | O | X |  |
| Muraenidae | *Gymnothorax eurostus* | O | X | X | O |  |
|  | *Gymnothorax javanicus* | O | **X** | O | O |  |
|  | *Gymnothorax meleagris* |  | X | X | X |  |
| Synodontidae | *Synodus capricornis* |  | **X** | **X** | O |  |
| Holocentridae | *Myripristis berndti* | X | X | X | **X** |  |
|  | *Myripristis tiki* | O | **X** | **X** | O |  |
|  | *Myrpristis amaena* | O | X | X |  |  |
|  | *Neoniphon opercularis* | **X** |  | **X** |  | **X** |
|  | *Neoniphon sammara* | X | O | **X** |  |  |
|  | *Sargocentron diadema* |  | X | X | O |  |
|  | *Sargocentron punctatissimum* |  | X | O | O |  |
|  | *Sargocentron spiniferum* | X | X | X |  |  |
|  | *Sargocentron tiere* | X | X | X | X |  |
| Aulostomidae | *Aulostomus chinensis* | X | **X** | X | X |  |
| Fistulariidae | *Fistularia commersonii* | X | X | X | X |  |

Table S4. Continued.

| Family | Taxon | Ducie | Henderson | Oeno | Pitcairn | New record for all islands |
| --- | --- | --- | --- | --- | --- | --- |
| Scorpaenidae | *Pterois antennata* |  | X | O | X |  |
|  | *Scorpaenopsis possi* |  | X |  | O |  |
| Caracanthidae | *Caracanthus maculatus* |  | **X** | X | X |  |
| Serranidae | *Cephalopholis argus* | X | O | X | O |  |
|  | *Cephalopholis spiloparaea* |  | **X** | **X** | X |  |
|  | *Cephalopholis urodeta* | **X** | X | X | X |  |
|  | *Epinephelus fasciatus* | X | X | X | X |  |
|  | *Epinephelus hexagonatus* | O | X | X | X |  |
|  | *Epinephelus merra* | **X** | O |  |  |  |
|  | *Epinephelus socialis* | O | O |  | X |  |
|  | *Epinephelus tauvina* | X | X | X | X |  |
|  | *Pseudanthias mooreanus* |  | X | X | O |  |
|  | *Pseudanthias ventralis* | O | X |  | O |  |
|  | *Variola louti* | X | X | X | X |  |
| Cirrhitidae | *Cirrhitops hubbardi* | **X** | X | X | X |  |
|  | *Cirrhitus pinnulatus* |  | X |  | X |  |
|  | *Neocirrhites armatus* | **X** | **X** | X | X |  |
|  | *Paracirrhites arcatus* | **X** | X | X | X |  |
|  | *Paracirrhites forsteri* | X | X | **X** | X |  |
|  | *Paracirrhites hemistictus* | X | X | X | X |  |
| Priacanthidae | *Heteropriacanthus cruentatus* | X | X | O | X |  |
| Apogonidae | *Ostorhinchus angustatus* |  |  | O | X |  |

Table S4. Continued.

| Family | Taxon | Ducie | Henderson | Oeno | Pitcairn | New record for all islands |
| --- | --- | --- | --- | --- | --- | --- |
| Carangidae | *Carangoides orthogrammus* | **X** | X |  | X |  |
|  | *Caranx ignobilis* | O | X | X |  |  |
|  | *Caranx lugubris* | X | X | X | X |  |
|  | *Caranx melampygus* | X | X | X | X |  |
|  | *Decapterus species* |  |  | X | **X** |  |
|  | *Pseudocaranx dentex* |  | O | O | X |  |
|  | *Scomberoides lysan* |  | **X** | **X** |  | **X** |
|  | *Seriola lalandi* | X | X | O | O |  |
| Carangidae | *Seriola rivoliana* | O | **X** | **X** | **X** |  |
| Lutjanidae | *Aphareus furca* |  | X | X | X |  |
|  | *Lutjanus bohar* | X | X | X | O |  |
|  | *Lutjanus kasmira* | O | O | X | X |  |
| Caesionidae | *Pterocaesio tile* | **X** | O |  | X |  |
| Lethrinidae | *Gnathodentex aureolineatus* | X | X | X | X |  |
|  | *Lethrinus olivaceous* |  | X | **X** |  |  |
|  | *Monotaxis grandoculis* | X | X | X | X |  |
| Mullidae | *Mulloidichthys vanicolensis* | **X** | X | X | X |  |
|  | *Parupeneus cyclostomus* | X | X | X | X |  |
|  | *Parupeneus insularis* | X | X | X | X |  |
|  | *Parupeneus multifasciatus* | X | X | X | X |  |
|  | *Parupeneus pleurostigma* | O |  | **X** | X |  |
| Pempheridae | *Pempheris oualensis* | O | X |  | X |  |

Table S4. Continued.

| Family | Taxon | Ducie | Henderson | Oeno | Pitcairn | New record for all islands |
| --- | --- | --- | --- | --- | --- | --- |
| Kyphosidae | *Kyphosus sp* | X | X | X | X |  |
| Chaetodontidae | *Chaetodon auriga* | X | X | X | O |  |
|  | *Chaetodon flavirostris* | X | X | X | **X** |  |
|  | *Chaetodon lunula* | **X** | X | X | X |  |
|  | *Chaetodon mertensii* | O | X | X | X |  |
|  | *Chaetodon ornatissimus* | X | X | X | X |  |
|  | *Chaetodon pelewensis* | X | X | X | X |  |
|  | *Chaetodon quadrimaculatus* | X | X | X | X |  |
|  | *Chaetodon reticulatus* | X | X | **X** | X |  |
|  | *Chaetodon smithi* | O |  | **X** | X |  |
|  | *Chaetodon ulietensis* | X |  |  |  |  |
|  | *Chaetodon unimaculatus* | X | X | **X** | O |  |
|  | *Forcipiger flavissimus* | X | X | **X** | X |  |
|  | *Forcipiger longirostris* | **X** | X | **X** | X |  |
|  | *Hemitaurichthys multispinosus* | **X** |  |  | O |  |
|  | *Heniochus chrysostomus* |  |  | X | X |  |
|  | *Heniochus monoceros* | **X** | X | X | **X** |  |
| Pomacanthidae | *Centropyge flavissima* | X | X | X | X |  |
|  | *Centropyge hotumatua* | X | X | X | X |  |
|  | *Centropyge loricula* | X | X | X | X |  |
|  | *Genicanthus spinus* | **X** | O | O | X |  |
|  | *Pomacanthus imperator* |  | X |  | X |  |

Table S4. Continued.

| Family | Taxon | Ducie | Henderson | Oeno | Pitcairn | New record for all islands |
| --- | --- | --- | --- | --- | --- | --- |
| Pomacentridae | *Abudefduf sordidus* | O | O |  | X |  |
|  | *Chromis acares* | **X** | **X** | X | **X** |  |
|  | *Chromis agilis* | X | X | X | X |  |
|  | *Chromis bami* | X | X | X | X |  |
|  | *Chromis pamae* | **X** | X | X | X |  |
|  | *Chromis vanderbilti* | X | **X** | **X** | X |  |
|  | *Chromis xanthura* | **X** | **X** | X | X |  |
|  | *Chrysiptera galba* | X | X | X | X |  |
|  | *Chrysiptera glauca* |  |  | **X** | X |  |
|  | *Dascyllus flavicaudus* | **X** | X | X | O |  |
|  | *Dascyllus trimaculatus* |  |  | **X** | O |  |
|  | *Plectroglyphidodon dickii* |  | **X** | **X** |  | **X** |
|  | *Plectroglyphidodon flaviventris* |  |  | **X** |  | **X** |
|  | *Plectroglyphidodon imparipennis* | X | X | **X** | X |  |
|  | *Plectroglyphidodon johnstonianus* | **X** | X | X | X |  |
|  | *Plectroglyphidodon leucozonus* |  | O |  | X |  |
|  | *Plectroglyphidodon phoenixensis* | **X** | O | O | **X** |  |
|  | *Pomochromis fuscidorsalis* | X | X | X | X |  |
|  | *Stegastes emeryi* | X | X | X | X |  |
|  | *Stegastes fasciolatus* | X | X | X | X |  |
| Labridae | *Anampses caeruleopunctatus* | X | X | X | X |  |
|  | *Anampses femininus* |  |  | O | X |  |

Table S4. Continued.

| Family | Taxon | Ducie | Henderson | Oeno | Pitcairn | New record for all islands |
| --- | --- | --- | --- | --- | --- | --- |
|  | *Anampses twistii* |  |  | **X** | O |  |
|  | *Bodianus axillaris* |  | X | X | X |  |
|  | *Bodianus bilunulatus* | X | O |  |  |  |
|  | *Cheilinus trilobatus* |  |  | **X** |  | **X** |
|  | *Cheilinus undulatus* |  | X |  |  |  |
|  | *Cheilio inermis* |  |  |  | X |  |
|  | *Cirrhilabrus scottorum* |  | **X** | X | X |  |
|  | *Coris aygula* | X | X | X | X |  |
|  | *Coris roseoviridis* | **X** | **X** | X | X |  |
|  | *Gomphosus varius* | X | X | X | X |  |
|  | *Halichoeres margaritaceus* |  | **X** | O | X |  |
|  | *Halichoeres melasmapomus* |  | **X** |  | O |  |
|  | *Halichoeres ornatissimus* |  | **X** | **X** | **X** | **X** |
|  | *Hemigymnus fasciatus* | X | X | X | X |  |
|  | *Hologymnosus annulatus* |  | **X** | X | X |  |
|  | *Iniistius celebicus* |  |  |  | **X** |  |
|  | *Labroides bicolor* | **X** |  | X | X |  |
|  | *Labroides dimidiatus* | X | X | X | X |  |
|  | *Labroides rubrolabiatus* | X | X | X | X |  |
|  | *Labropsis polynesica* | **X** |  |  |  | **X** |
|  | *Macropharyngodon meleagris* | O | **X** | X | X |  |
|  | *Novaculichthys taeniourus* |  |  | X | **X** |  |

Table S4. Continued.

| Family | Taxon | Ducie | Henderson | Oeno | Pitcairn | New record for all islands |
| --- | --- | --- | --- | --- | --- | --- |
|  | *Oxycheilinus unifasciatus* | X | X | **X** | X |  |
|  | *Pseudocheilinus citrinus* | X | X | X | X |  |
|  | *Pseudocheilinus octotaenia* | **X** | X | **X** | X |  |
|  | *Pseudocheilinus tetrataenia* | X | X | X | X |  |
|  | *Pseudojuloides atavai* | X | **X** | X | X |  |
|  | *Pseudojuloides cerasinus* |  | **X** | **X** | **X** | **X** |
|  | *Pseudolabrus fuentesi* |  |  |  | X |  |
|  | *Stethojulis bandanensis* | **X** | O | X | X |  |
|  | *Thalassoma heiseri* | X | X | X | O |  |
|  | *Thalassoma lutescens* | X | X | X | X |  |
|  | *Thalassoma purpureum* | X | X | X | X |  |
|  | *Thalassoma trilobatum* | **X** | X | O | X |  |
| Scaridae | *Calotomus carolinus* | **X** | **X** | **X** | X |  |
|  | *Chlorurus frontalis* | X |  | X | O |  |
|  | *Chlorurus microrhinos* | X | O | X | O |  |
|  | *Chlorurus sordidus* | X |  | X |  |  |
|  | *Leptoscarus vaigiensis* | **X** | **X** |  | X |  |
|  | *Scarus altipinnis* | X | **X** | **X** | X |  |
|  | *Scarus forsteni* | X | X | X | X |  |
|  | *Scarus frenatus* | O | **X** | X |  |  |
|  | *Scarus ghobban* | O |  |  | **X** |  |
|  | *Scarus longipinnis* |  | X | **X** | X |  |

Table S4. Continued.

| Family | Taxon | Ducie | Henderson | Oeno | Pitcairn | New record for all islands |
| --- | --- | --- | --- | --- | --- | --- |
|  | *Scarus schlegeli* | **X** |  |  |  | **X** |
| Pinguipedidae | *Parapercis millepunctata* |  | **X** |  | O |  |
|  | *Parapercis schauinslandi* |  | **X** |  | O |  |
| Blenniidae | *Cirripectes variolosus* | X | X | **X** | X |  |
|  | *Exalias brevis* |  | X | **X** |  |  |
|  | *Plagiotremus tapeinosoma* | **X** | X | X | X |  |
| Gobiidae | *Gnatholepis.c.australis* | **X** | **X** | X | O |  |
| Ptereleotridae | *Nemateleotris magnifica* | **X** | X | X | X |  |
| Zanclidae | *Zanclus cornutus* | X | X | X | X |  |
| Acanthuridae | *Acanthurus achilles* | X | X | **X** |  |  |
|  | *Acanthurus guttatus* |  | O |  | X |  |
|  | *Acanthurus leucopareius* | X | X | X | X |  |
|  | *Acanthurus leucopareius/nigroris/nigrofuscus* | O | X | X | X |  |
|  | *Acanthurus nigrofuscus* | **X** | X | **X** | X |  |
|  | *Acanthurus nigroris* |  | X | **X** | **X** |  |
|  | *Acanthurus nubilus* | X | X | **X** | O |  |
|  | *Acanthurus olivaceus* |  | **X** |  |  | **X** |
|  | *Acanthurus thompsoni* | X | X | X | O |  |
|  | *Acanthurus triostegus* | O | X | O | X |  |
|  | *Ctenochaetus flavicauda* | X | X | X | X |  |
|  | *Ctenochaetus hawaiiensis* | X | X | **X** | O |  |
|  | *Ctenochaetus striatus* | **X** | O | X | X |  |

Table S4. Continued.

| Family | Taxon | Ducie | Henderson | Oeno | Pitcairn | New record for all islands |
| --- | --- | --- | --- | --- | --- | --- |
|  | *Naso brevirostris* | X | X | **X** |  |  |
|  | *Naso caesius* |  | X |  | O |  |
|  | *Naso hexacanthus* | X | X | **X** | O |  |
|  | *Naso lituratus* | **X** | **X** | X | X |  |
|  | *Naso unicornis* | **X** | X | X | X |  |
|  | *Zebrasoma rostratum* | X | X | **X** | X |  |
|  | *Zebrasoma scopas* |  | O |  | X |  |
|  | *Zebrasoma veliferum* | **X** | X | **X** | X |  |
| Sigandae | *Siganus argenteus* |  |  | X | X |  |
| Sphyraenidae | *Sphyraena qenie* |  | **X** | **X** |  | **X** |
| Scombridae | *Gymnosarda unicolor* |  | **X** |  | X |  |
| Balistidae | *Balistoides viridescens* | **X** | X | X | X |  |
|  | *Pseudobalistes fuscus* | O |  | X | O |  |
|  | *Rhinecanthus lunula* | X | O | **X** | X |  |
|  | *Rhinecanthus rectangulus* | O | X | X | X |  |
|  | *Sufflamen bursa* | X | X | X | X |  |
|  | *Sufflamen frenatus* |  | X | X | **X** |  |
|  | *Xanthichthys mento* | **X** |  | O | X |  |
| Monacanthidae | *Aluterus scriptus* | **X** | X |  | O |  |
|  | *Cantherhines dumerilii* | X | X | **X** | X |  |
|  | *Cantherhines longicaudus* |  | **X** |  | **X** | **X** |
|  | *Cantherhines sandwichiensis* | X | X | **X** | X |  |

Table S4. Continued.

| Family | Taxon | Ducie | Henderson | Oeno | Pitcairn | New record for all islands |
| --- | --- | --- | --- | --- | --- | --- |
|  | *Pervagor aspricaudus* |  |  | **X** |  | **X** |
| Ostraciidae | *Lactoria diaphana* | O |  |  | **X** |  |
| Tetraodontidae | *Arothron meleagris* | X | X | X | X |  |
|  | *Canthigaster coronata* |  | **X** | **X** |  | **X** |
|  | *Canthigaster janthinoptera* |  |  | X | O |  |
| Diodontidae | *Diodon holocanthus* | **X** | X | O | O |  |
|  | *Diodon hystrix* | X |  | O | X |  |

References

Irving RA, Jamieson J, Randall JE (1995) Initial checklist of fishes from Henderson Island, Pitcairn Group. Biological Journal of the Linnean Society 56: 329-338.

Randall JE (1999) Report on fish collections from the Pitcairn Islands. Atoll Research Bulletin 461: 1-53.
