# Supplementary material for: The Real Bounty: Marine Biodiversity in the Pitcairn Islands
Source: PLoS One. 2014 Jun 25;9(6):e100142. doi: 10.1371/journal.pone.0100142 (PMC4070931; doi:10.1371/journal.pone.0100142)
Supplement: Table S5 — Fishes observed in deep habitats of the Pitcairn islands, using National Geographic's Drop-Cams. (DOCX) [file pone.0100142.s005.docx]

Table S5. Fishes observed in deep habitats of the Pitcairn islands, using National Geographic’s Drop-Cams.

| Family | Taxon | Ducie | Henderson | Oeno | Pitcairn | 40-mile reef | Observed depth range (m) | Deep habitat type |
| --- | --- | --- | --- | --- | --- | --- | --- | --- |
| Squalidae | *Squalus* sp. |  |  | × | × |  | 805-835 | Rock, sand |
| Hexanchidae | *Hexanchus griseus* | × | × |  |  |  | 647-795 | Rocky slope with sediment |
| Carcharhinidae | *Charcharhinus amblyrhynchos* | × | × |  | × |  | 0-382 | Rock, sand, rocky slope with sediment |
| Pseudotriakidae | *Pseudotriakis microdon* | × | × | × |  |  | 795-1060 | Sand |
| Gempylidae | *Rexea* sp.1 | × |  |  | × |  | 795-805 | Rock, sand |
|  | *Rexea* sp.2 |  |  |  | × |  | 835 | Rock, sand |
|  | *Ruvettus* sp. |  | × | × |  |  | 835-1060 | Fine sand |
| Polymixidae | *Polymixia* sp. | × | × | × | × |  | 795-1060 | Rock, sand |
| Argentinidae | Unidentified Argentinidae |  |  |  | × |  | 805 | Rock, sand |
| Scombridae | *Gymnosarda unicolor* |  |  |  | × |  | 0-805 | Rock, sand, rocky slope with sediment |

Table S5. Continued.

| Family | Taxon | Ducie | Henderson | Oeno | Pitcairn | 40-mile reef | Observed depth range (m) | Deep habitat type |
| --- | --- | --- | --- | --- | --- | --- | --- | --- |
| Serranidae | *Epinephelus fasciatus* |  |  |  |  | × | 0-78 | Deep coral reef |
|  | *Epinephelus tuamotensis* | × | × | × |  | × | 78-312 | Rock, sand, deep coral reef, rocky slope with sediment |
|  | *Pseudanthias* sp. nov.1 |  | × |  |  |  | 216-230 | Rocky slope with sediment |
|  | *Pseudanthias* sp. nov.2 |  | × |  |  |  | 216 | Rocky slope with sediment |
|  | *Pseudanthias* sp. nov.3 |  |  | × |  |  | 288 | Rocky slope with sediment |
|  | *Variola louti* |  |  |  |  | × | 142 | Rock, sand, deep coral reef |
| Carangidae | *Caranx lugubris* | × | × | × |  | × | 0-312 | Rock, sand, deep coral reef, rocky slope with sediment |
|  | *Seriola lalandi* | × |  |  |  | × | 0-312 | Rocky slope with sediment |
|  | *Seriola rivoliana* |  | × | × |  | × | 0-288 | Rock, sand, deep coral reef, rocky slope with sediment |
| Gobiidae | Unidentified Gobiidae spp. | × | × |  |  |  | 216-382 | Rocky slope with sediment |

Table S5. Continued.

| Family | Taxon | Ducie | Henderson | Oeno | Pitcairn | 40-mile reef | Observed depth range (m) | Deep habitat type |
| --- | --- | --- | --- | --- | --- | --- | --- | --- |
| Chaetodontidae | *Chaetodon pelewensis* |  |  |  |  | × | 78 | Deep coral reef |
|  | *Chaetodon* sp. nov.1 | × |  |  |  |  | 234 | Rock, sand |
| Pomacentridae | *Chromis pamae* |  |  |  |  | × | 78 | Deep coral reef |
|  | Unidentified Pomacentridae 1 | × |  |  |  |  | 234 | Rock, sand |
|  | Unidentified Pomacentridae 2 |  |  |  |  | × | 78 | Deep coral reef |
| Monacanthidae | Unidentified Monacanthidae | × | × |  | × |  | 216-234 | Rocky slope with sediment |
| Pinguipedidae | *Parapercis* sp. | × |  |  |  |  | 234 | Rock, sand |
| Muraenidae | *Gymnothorax eurostus* |  |  |  |  | × | 78 | Deep coral reef |
|  | *Gymnothorax meleagris* |  |  |  |  | × | 78 | Deep coral reef |
|  | *Gymnothorax nudimover* |  | × |  |  |  | 207 | Rocks with sand |

Table S5. Continued.

| Family | Taxon | Ducie | Henderson | Oeno | Pitcairn | 40-mile reef | Observed depth range (m) | Deep habitat type |
| --- | --- | --- | --- | --- | --- | --- | --- | --- |
| Caesonidae | *Pterocaesio* sp. nov. |  | × |  |  |  | 230 | Rocky slope with sediment |
| Antennariidae | *Antennarius* sp. |  | × |  |  |  | 230 | Rocky slope with sediment |
| Lutjanidae | *Aprion virescens* |  | × | × |  |  | 216-230 | Rocky slope with sediment |
|  | *Etelis carbunculus* |  |  | × | × |  | 282-538 | Rocky slope with sediment |
|  | *Lutjanus bohar* |  |  |  |  | × | 78 | Deep coral reef |
|  | Lutjanidae sp. nov? |  | × | × |  |  | 230-288 | Rocky slope with sediment |
| Liopropominae | *Liopropoma* sp. |  | × | × |  |  | 216-230 | Rocky slope with sediment |
| Synodontidae | *Synodus* sp.1 |  | × |  |  |  | 382 | Rocky slope with sediment |
|  | *Synodus* sp.2 |  |  |  |  | × | 142 | Sand, coarse gravel |
| Macrouridae | *Caelorinchus* cf. sp. |  | × |  |  |  | 1060 | Fine sand |

Table S5. Continued.

| Family | Taxon | Ducie | Henderson | Oeno | Pitcairn | 40-mile reef | Observed depth range (m) | Deep habitat type |
| --- | --- | --- | --- | --- | --- | --- | --- | --- |
| Syphanobranchidae | *Syphanobranchus* sp. |  |  | × |  |  | 629 | Rocky slope with sediment |
| Carapidae | Unidentified Carapidae |  |  | × |  |  | 538 | Rocky slope with sediment |
| Apogonidae | *Apogon* sp. nov. |  |  | × |  |  | 538 | Rocky slope with sediment |
| Nettastomatidae | Unidentified Nettastomatidae |  |  | × |  |  | 1359-1585 | Rock, sand |
| Moridae | *Antimora* sp. |  |  | × |  |  | 1585 | Rock, sand |
| Malacanthidae | *Malacanthus brevirostris* |  |  |  |  | × | 142 | Rock, sand |
| Labridae | *Bodianus unimaculatus* |  |  |  |  | × | 142 | Rock, sand |
|  | *Labroides dimidiatus* |  |  |  |  | × | 10-78 | Deep coral reef |
|  | Unidentified Labridae |  |  |  |  | × | 78-142 | Rock, sand, deep coral reef |
| Balistidae | *Sufflamen fraenatum* |  |  |  |  | × | 20-142 | Rock, sand, deep coral reef |

Table S5. Continued.

| Family | Taxon | Ducie | Henderson | Oeno | Pitcairn | 40-mile reef | Observed depth range (m) | Deep habitat type |
| --- | --- | --- | --- | --- | --- | --- | --- | --- |
| Pomacanthidae | *Centropyge hotumatua* |  |  |  |  | × | 78 | Deep coral reef |
|  | *Centropyge* sp. nov. |  |  |  |  | × | 78-142 | Rock, sand, deep coral reef |
|  | *Genicanthus spinus* |  |  |  |  | × | 42-78 | Deep coral reef |
| Kyphosidae | *Kyphosus* sp. |  |  |  |  | × | 78 | Deep coral reef |
| Aulostomidae | *Aulostomus chinensis* |  |  |  |  | × | 78 | Deep coral reef |
| Acanthuridae | *Acanthurus* sp. |  |  |  |  | × | 78 | Deep coral reef |
