# Supplementary material for: The Real Bounty: Marine Biodiversity in the Pitcairn Islands
Source: PLoS One. 2014 Jun 25;9(6):e100142. doi: 10.1371/journal.pone.0100142 (PMC4070931; doi:10.1371/journal.pone.0100142)
Supplement: Table S6 — Invertebrates observed in deep habitats of the Pitcairn islands, using National Geographic's Drop-Cams. (DOCX) [file pone.0100142.s006.docx]

Table S6. Invertebrates observed in deep habitats of the Pitcairn islands, using National Geographic’s Drop-Cams.

| Taxon | Ducie | Henderson | Oeno | Pitcairn | 40-mile reef | Observed depth range (m) | Deep habitat type |
| --- | --- | --- | --- | --- | --- | --- | --- |
| SPONGES |  |  |  |  |  |  |  |
| Unidentified sponges |  | × |  |  |  | 216 | Rocky slope with sediment |
| CNIDARIANS |  |  |  |  |  |  |  |
| Unidentified whip coral | × | × |  |  |  | 207-234 | Rocks, sand, rocky slope with sediment |
| Unidentified gorgonian sp1 |  | × |  |  |  | 230 | Rocky slope with sediment |
| Unidentified gorgonian sp2 |  | × |  |  |  | 230 | Rocky slope with sediment |
| Unidentified gorgonian sp3 |  |  |  | × |  | 282 | Rocky slope with sediment |
| Unidentified gorgonian sp4 |  |  |  | × |  | 282 | Rocky slope with sediment |
| *Pocillopora* sp. |  |  |  |  | × | 78-142 | Deep coral reef, sand |
| *Porites* *deformis* |  |  |  |  | × | 78-142 | Deep coral reef, sand |
| *Sinularia* sp. |  |  |  |  | × | 78-142 | Deep coral reef, sand |

Table S6. Continued.

| Taxon | Ducie | Henderson | Oeno | Pitcairn | 40-mile reef | Observed depth range (m) | Deep habitat type |
| --- | --- | --- | --- | --- | --- | --- | --- |
| POLYCHAETES |  |  |  |  |  |  |  |
| Serpulidae |  |  |  | × |  | 598 | Rocky slope with sediment |
| CRUSTACEANS |  |  |  |  |  |  |  |
| Mysidae | × |  | × | × |  | 312-805 | Rocks, sand, basalt slope, rocky slope with sediment |
| Amphipoda |  | × | × |  |  | 629-1585 | Fine sand, rocky slope with sediment, coarse sand with rocks |
| Galathidae | × | × | × |  |  | 216-598 | Rocks, sand, rocky slope with sediment |
| Paguridae | × | × |  |  |  | 234-652 | Rocks, sand, rocky slope with sediment |
| Parapaguridae |  |  | × | × |  | 538-598 | Rocky slope with sediment |
| Euphausiacea |  | × | × |  |  | 629-1585 | Fine sand, rocky slope with sediment, coarse sand with rocks |

Table S6. Continued.

| Taxon | Ducie | Henderson | Oeno | Pitcairn | 40-mile reef | Observed depth range (m) | Deep habitat type |
| --- | --- | --- | --- | --- | --- | --- | --- |
| Majidae |  |  | × |  |  | 629 | Rocky slope with sediment |
| MOLLUSKS |  |  |  |  |  |  |  |
| Unidentified squid | × |  |  | × |  | 598-805 | Rocks, sand, rocky slope |
| Unidentified Octopus |  |  |  | × |  | 598 | Rocky slope |
| Ophiuroidea | × |  |  |  |  | 234 | Rocks, sand |
| SEA URCHINS |  |  |  |  |  |  |  |
| *Diadema* sp. |  |  |  |  | × | 78-142 | Deep coral reef |
| TUNICATES |  |  |  |  |  |  |  |
| Unidentified gelatinous zooplankton |  |  |  | × |  | 598 | Rocky slope with sediment |
